# Alps to Apennines zircon roller coaster along the Adria microplate margin

**DOI:** 10.1038/s41598-018-20979-w

**Published:** 2018-02-09

**Authors:** J. Jacobs, G. Paoli, S. Rocchi, A. K. Ksienzyk, H. Sirevaag, M. A. Elburg

**Affiliations:** 1 Department of Earth Science, University of Bergen, P.O.Box 7800, 5020 Bergen, Norway; 2 Dipartimento di Scienze della Terra, Università di Pisa, Pisa, I-56126 Italy; 3 Department of Geology, University of Johannesburg, Auckland Park, 2006 Johannesburg, South Africa

## Abstract

We have traced the particle path of high-pressure metasedimentary rocks on Elba Island, Northern Apennines, with the help of a U-Pb-Hf detrital zircon study. One quarter of the analysed zircons are surprisingly young, 41-30 Ma, with a main age peak at ca. 32 Ma, indicating an unexpected early Oligocene maximum deposition age. These Oligocene ages with negative εHf indicate a volcanic source region in the central-southern Alps. Though young by geological means, these zircons record an extraordinary geodynamic history. They originated in a volcanic arc, during the convergence/collision of the the Adria microplate with Europe from ca. 65 to 30 Ma. Thereafter, the Oligocene zircons travelled ca. 400 km southward along the Adria margin and the accretionary prism to present-day Tuscany, where they were subducted to depths of at least 40 km. Shortly thereafter, they were brought to the surface again in the wake of hinge roll back of the Apennine subduction zone and the resulting rapid extensional exhumation. Such a zircon roller coaster requires a microplate that has back-to-back subduction zones with opposing polarities on two sides.

## Introduction

The Adria microplate is a Gondwana-derived terrane that drifted northward to eventually collide with Europe. The subduction of Europe underneath Adria started in late Cretaceous times and led to continental collision in the Oligocene-Miocene, resulting in the formation of the Alps^1,2^. In the meantime, to the south, the Adria plate was subducting to the NW underneath the Corsica-Sardinia block of the Europe plate, likely after an Eocene subduction flip from SE-directed subduction of Europe underneath Adria^2,3^. Thus, the Alps and Apennines evolved subsequently and largely as two independent orogenic systems along the northern and western side of the Adria microplate respectively, with contrasting subduction polarities. The Apennine subduction is a classic example for slab roll back, characterised by subduction-accretion, followed by extensional exhumation as a result of slab hinge retreat^1,4,5^.

**Figure 1:**
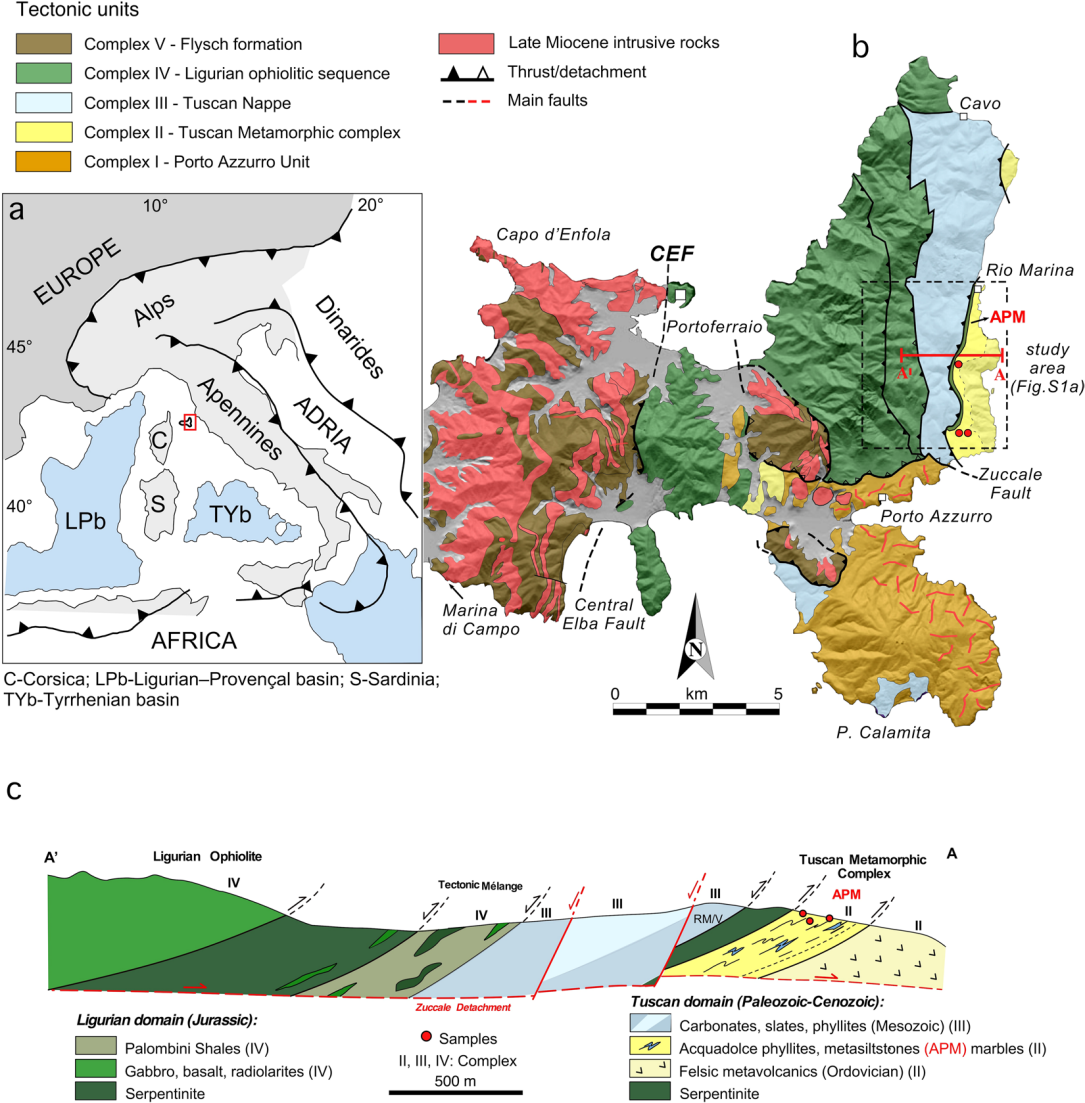
Geological setting of Elba Island. (**a**) Geodynamic sketch map of the central Mediterranean region. (**b**,**c**) Simplified geological map and schematic geological cross-section of eastern Elba Island, showing the stacked tectonic units and sample localities in the transition zone from the Tuscan Metamorphic Complex to the Ligurian Ophiolite (modified after^11,36,37^). *Abbreviations:* CEF: Central Elba Fault; RM: Rio Marina phyllites; V: Verruca quartzites. The figs were created using Canvas Draw 3 (version 3.0.3) http://www.canvasgfx.com/en/products/canvas-draw.

Elba Island forms the innermost part of the Northern Apennines, where the continental Tuscan domain (Adria Plate) and oceanic units (Ligurian domain of the Alpine Tethys) were tectonically juxtaposed and stacked during the Alpine-Apennine subduction-accretion-extension cycle^6,7^ (Fig. 1). In eastern Elba Island, the Tuscan Metamorphic Units record in part subduction to depths of up to 40 km, followed by Miocene exhumation in an eastward retreating subduction framework^2,4,5,8^. They also include Oligocene metaturbidites (Pseudomacigno Formation) that were deposited in the eastward propagating Apennine foredeep. These units thus record the subduction–exhumation history of the Adria Plate margin and are therefore targeted here to gain a better understanding for the rate of sediment supply-subduction-exhumation processes.

## Elba Island – Tectonic setting

The tectono-stratigraphy of Elba Island is described in terms of five distinct tectono-metamorphic complexes^9^ (Fig. 1). The tectono-stratigraphically lowermost schist complex represents the Paleozoic basement of the Adria plate margin, being characterized by detrital zircons with African provenance^10^. Complexes II and III derive from the western (Tuscan) continental margin of Adria, whilst complexes IV and V represent Ligurian oceanic units from the Alpine Tethys^9,11^.

Complex II includes metavolcanic rocks and their erosional products (ca. 460 Ma)^10^, overlain by the Acquadolce unit^8,9,11–13^, which is the subject of this study. The Acquadolce unit consists of ca. 20 m of marbles and calc-schists, overlain by a thick sequence of phyllites and metasiltstones (hereafter Acquadolce phyllites and metasiltstones - APM). The APM includes discontinuous layers of calc-schists characterised by scattered lenses of metabasites with high-P paragenesis, i.e. glaucophane formed at epidote blueschist facies conditions, recording maximum pressure of 0.9–1.0 GPa at ca. 330–350 °C^8^. The APM are highly deformed, displaying asymmetric isoclinal folds as well as sheath folds and oblique folds in calc-schists lenses (Supplementary File 1). Muscovite grown on the foliation planes in the calc-schists provided a ^40^Ar-^39^Ar age of 19.68 ± 0.15 Ma^14^, which is so far the best age estimate for the stacking-related metamorphic overprint. The deposition age of the APM is still unclear: it is most commonly considered of Cretaceous age^11,13^, although similarities with the Pseudomacigno formation of mainland Tuscany may suggest a much younger age^12^.

## Samples and Methods

**Figure 2:**
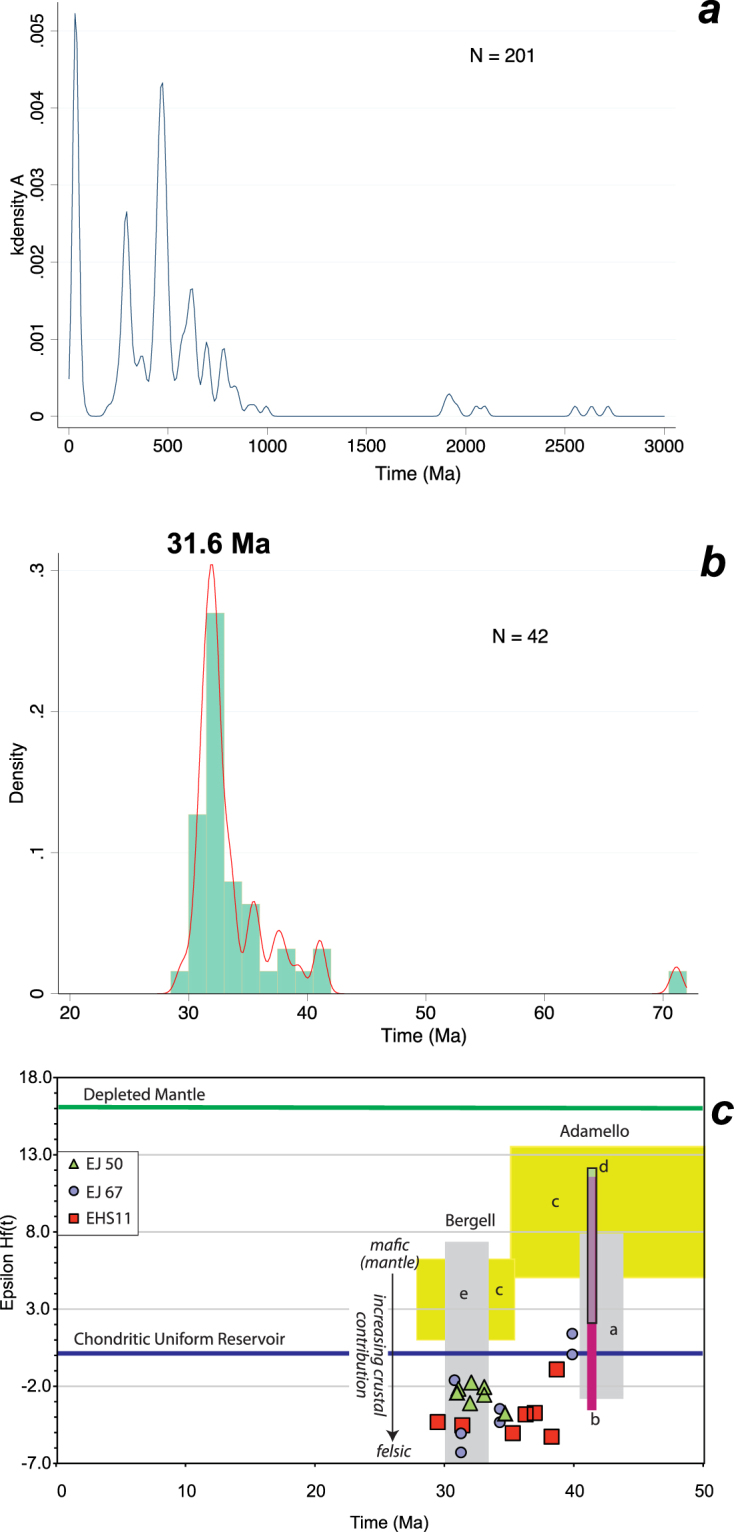
U-Pb and Lu-Hf zircon results. (**a**) Kernel density distribution of 201 concordant U-Pb zircon analyses of three analysed samples; Kernel half width 15 Ma. (**b**) Zircon age distribution and kernel density distribution of 42 concordant Oligocene zircons with a main age peak at 31.6 Ma; bin width 1.5 Ma. (**c**) Initial epsilon Hf versus age for the Oligocene zircons. Literature data for Hf isotopes on zircon: a^28^; b^26^; c^27^; d^38^; e: calculated from the whole rock Nd isotope data^29^, using the terrestrial array^30^.

Three samples of the APM underwent a U-Pb + Lu-Hf detrital zircon characterisation. Two samples, EJ50 and EJ67, were collected to the west of the Capo d´Arco residence in the southern part of the study area, whilst the third sample was collected along the road to Ortano (Fig. 2, Supplementary File 1). All three samples are from silty to sandy parts of the APM and were found to contain abundant zircon grains that were separated using conventional mineral separation techniques. Ca. 300 zircons per sample were handpicked, mounted in epoxy and sectioned into half by grinding and polishing. After cathodoluminescence (SEM-CL) imaging, zircons were first analysed for U-Pb by LA-ICP-MS and/or SHRIMP; in a second step, zircons underwent Lu-Hf characterisation (Supplementary File 2). A SEM-CL zircon typology study was carried out on 55 dated grains. Petrographic and geochemical analyses were also performed to define the nature of the studied metasedimentary rocks.

### Petrography and geochemistry

The phyllites and metasiltstones are highly foliated and consist of alternating very fine- and medium-grained layers (Supplementary File 1). The former are dominated by quartz, sericite, chlorite and opaque minerals, the latter contain detrital K-feldspar, plagioclase (An_30–35_) and calcic pyroxene (diopside with Mg# ~0.65–0.70) along with accessory titanite, rutile, zircon, allanite and apatite. Detrital minerals are accompanied by actinolite (Mg# ~0.65–0.70), chlorite and albite, representing a greenschist facies assemblage after a mafic protolith. Whole-rock chemical compositions are characterized by SiO_2_ = 54–65 wt%, elevated MgO (>3 wt%), and CaO (~5–13 wt%), as well as high contents of compatible elements like Ni and Cr, up to 60 and 150 ppm, respectively (Supplementary File 1). The relatively low loss on ignition (≤1 wt.%) implies that the enrichment in calcium is not the result of significant carbonate content. The petrology and geochemistry of the samples thus point to a mixed felsic and mafic, probably volcanic provenance.

### U-Pb-Hf data of phyllites and metasiltstones

The three analysed samples are dominated by relatively small (<200 µm), short-prismatic zircons. Many zircons are clear and idiomorphic, some have rounded tips, whilst others are zircon fragments. In CL, most zircons show simple or oscillatory growth zoning (Supplementary File 3a), indicating an igneous origin for the vast majority of zircons, while typical metamorphic zircons are scarce. Sample EJ50 provided 112 concordant LA-ICPMS U-Pb zircon data. SHRIMP U-Pb zircon analyses of samples EJ50, EJ67 and HSE11 provided another 89 concordant single grain age data (Supplementary Files 3b,c). All three samples show very similar single grain age distributions (Fig. 2 and Supplementary File 3). Three main age peaks occur at ca. 32 Ma, 280 Ma and 480 Ma, which together make up two-thirds of all zircon ages. The remaining zircon ages are mostly late Neoproterozoic; a few Paleoproterozoic and Archean ages also occur.

The youngest zircon population is unexpectedly young, Eocene-Oligocene in age, and makes up approximately a quarter of the entire zircon population. This age group has an asymmetric peak of 31.6 Ma and an older age tail up to ca. 41 Ma. These young zircons typically have Th/U ranging from 0.12–1.0 and are characterised by oscillatory zoning, pointing out their igneous nature. A mostly volcanic, rather than plutonic, origin is suggested by the preservation of tiny edges of zircon grains. The magmatic affinity has been investigated by means of a typology analysis^15^ using CL images^16^ of 55 dated crystals, indicating a most likely origin from a calc-alkaline association, along with minor occurrences of shapes typical of peraluminous crustal products.

With the exception of one age of ca. 70 Ma, our samples lack Mesozoic and Paleocene age components. A significant age peak at ca. 280 Ma makes up 18% of the age population and relates to a late Variscan igneous provenance. The largest age peak with over a quarter of all ages is of Ordovician age, ca. 480 Ma, and most likely relates to voluminous Ordovician felsic magmatism at the active northern Gondwana margin^10,17^. The Neoproterozoic to Archean ages correlates to an African heritage and times when Adria had a peri-Gondwana position. Zircons with Th/U < 0.1, ca. 5% of the entire population, may be of metamorphic origin and are almost entirely related to Cambrian-Neoproterozoic ages.

Lu-Hf measurements for Oligocene zircons show significant scatter (Fig. 2c), from initial εHf of −7 to +1, with a mean of −4, indicating the involvement of old, probably Meso-Paleoproterozoic crust.

### Oligocene zircon provenance

The Oligocene zircons are interpreted as igneous zircons because they are euhedral, oscillatory zoned and have typical Th/U from 0.12–1.0. Furthermore, our zircon SEM-CL typology study indicates a calc-alkaline origin; the majority of the zircons are thus most likely related to Alpine-Apennine convergent tectonics. The overall detrital mineralogy and geochemistry indicate an andesitic to mafic source material of likely volcanic nature. The age of deposition must be younger than the youngest detrital zircons that the unit contains. The youngest concordant single zircon ages from the phyllites and metasiltstones are 29 ± 3 Ma (2σ) analysed by LA-ICP-MS, and 31 ± 2 Ma (2σ) analysed by SHRIMP. A more robust estimate of the maximum deposition age is the age of the youngest detrital zircon age component (i.e. group of zircons), which, for both LA-ICP-MS and SHRIMP analyses combined, is 31.6 ± 0.5 Ma (2σ). This is the best estimate for the maximum deposition age of the APM, which is much younger than previously assumed^11,13^, and makes it coeval with the more felsic foredeep deposits of the unmetamorphosed Macigno or the metamorphic Pseudomacigno units on mainland Tuscany^12^. Our new data show that these metasediments were deposited after ca. 32 Ma but before ca. 20 Ma, the age of the greenschist, stacking-related metamorphism^14^. Thus, they reveal very rapid cycling from volcanism to lateral fluvial translation, followed by subduction and extensional exhumation.

The potential source regions for the young Eocene-Oligocene zircons (Supplementary File 4) include the European plate, on the northern/western side of the Alps-Apennine system and/or the Adria Plate on the eastern/southern side^18^. Potential source regions of the European plate that were exposed by 30 Ma include magmatic active zones in Corsica-Sardinia and Provence-Esterel or rocks from the Alpine foreland basins, such as the Taveyanne Sandstones. The Cenozoic calc-alkaline igneous activity on Corsica-Sardinia ranges from 38 to 12 Ma, but with the climax around 20 Ma, thus postdating the zircon population in our rocks^19^. The same accounts for Provence-Esterel where the igneous activity starts at 41 Ma, but the most represented ages are in the range 33–19 Ma, again younger than APM zircon ages^20^.

Potential sources on the Adria Plate include the Periadriatic igneous complexes associated with, from west to east, the Biella, Bergell and Adamello plutons in the Alps^18,21^. The plutons themselves are no potential sources, since they were not exhumed before 10 Ma^22–24^, nevertheless their ages are important in suggesting ages of potential volcanic activities and/or antecrystic zircons that volcanoes can erupt. The Biella volcanic complex in northern Western Alps lacks ages older than ca. 33 Ma^25^. The Bergell pluton in Central Alps, with ages clustering around ca. 30 Ma, is deeply unroofed and is proven to have supplied volcanic detritus since the beginning of late Oligocene times^23^. The Adamello pluton in the Southern Alps shows emplacement ages of ca. 44–31 Ma^26–28^, which very well overlap with our detrital zircon ages, though no evidence for a volcanic complex above the Adamello pluton is reported so far. Therefore, the Bergell complex appears as the most likely source, although a minor contribution from Adamello cannot be excluded, to explain ages between 33 and 41 Ma found in the APM.

The Hf isotopic composition of our dated zircons also matches the range of isotopic compositions of the felsic rocks of the Adamello massif (Schaltegger *et al*., 2009; Schoene *et al*., 2012) very well (Fig. 2D). The zircon data for the Adamello and Bergell mafic samples^27^ are more primitive than for the zircons we analysed. For the Bergell, the whole rock Nd isotope data^29^ shows that more felsic rocks are isotopically more enriched (i.e. more contaminated; lower εNd and εHf). If we calculate the Hf isotopic composition of zircon in equilibrium with the whole rock Nd isotopes, following the terrestrial array^30^, there is good overlap with the younger group of our zircons. The plutons volcanic roofs are fully eroded now^23^. Their erosional products, however, are seen in the Oligocene Aveto-Petrignacola Formation that includes conglomerates of volcanic origin^31^. Thus, our Oligocene zircons could either have been derived directly from the volcanic roof of Bergell-age volcanic complexes or their respective erosional derivates in Aveto. Their erosional products must have been fed longitudinally from the Alps into the Apennine foredeep basin^32^ and reached latitudes equivalent to present-day Elba Island.

### Paleozoic-Precambrian zircon provenance

**Figure 3:**
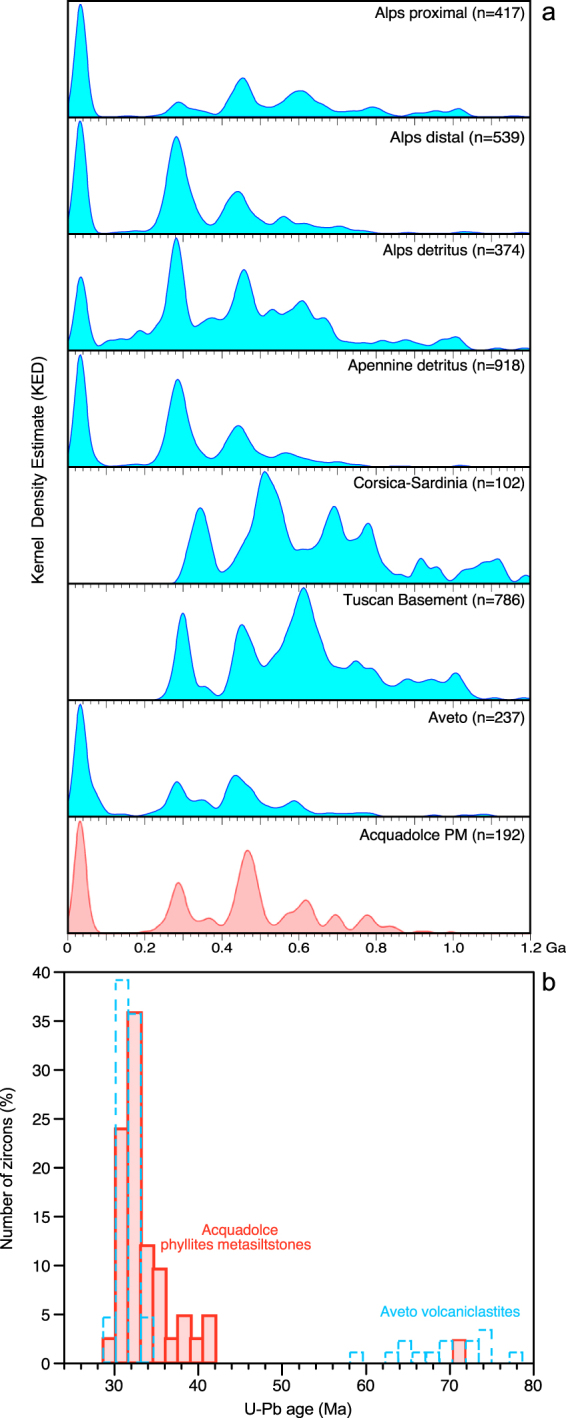
Comparison of the APM zircon age record with potential source regions. (**a**) Kernel Density Estimation (KDE^39^) plots for potential sources of Cenozoic and pre-Mesozoic zircons: APM plot is compared with the age distributions from potential basement sources: proximal and distal successions of the Adria foredeep^22^, Corsica-Sardinia^33^, Tuscan basement^10,17^, modern rivers draining the western-central-southern Alps and the northern Apennines^34^, volcaniclastic detritus of the Aveto unit^18^. (**b**) Comparison of the Cenozoic zircon age distributions of the APM and Aveto fm^18^.

Provenance of the pre-Mesozoic zircons (Fig. 3a) also argues for a source region in the Alps. If our zircon pool is compared with the age distribution spectra of various parts of the Alps, Apennines, and Corsica-Sardinia (Fig. 3a), then closest similarities are seen with the proximal and distal succession of the Adria foredeep^22^. In the Oligocene, the Tuscan basement^10,17^ was largely covered by sedimentary rocks and was therefore not available to contribute as a source for the sediments. The Eocene-Miocene deposits from Corsica-Sardinia have detrital zircon age distributions that lack a significant 250–300 Ma (Variscan) age component, but instead have a significant Grenville-age age group^33^, the latter of which is not present in our samples. The age distribution spectra (Fig. 3a) show that modern rivers draining the western-central-southern Alps and the northern Apennines have a proportion of Permian-Carboniferous (Variscan) zircons that is commonly larger than found in our samples, with the notable exception of sediments drained from the Ticino subdome of the Lepontine dome, dominated by Ordovician (Caledonian) zircon ages^34^. This suggests that crystalline detritus found in APM most likely derives from an area around the Bergell igneous complex. Our samples most likely represent a mixture of detritus dominated by a Central Alps provenance. Recycling through the Aveto-Petrignacola Oligocene sedimentary basin is also possible, owing to the comparable overall detrital zircon age distributions of Aveto and APM (Fig. 3). Nevertheless, in detail, some differences in the peak distributions and heights can be noted, such as the similar importance of Ordovician and Oligocene peaks in the APM, contrasting with the modest abundance of Ordovician zircons for two Aveto samples, and the strong dominance of Ordovician over Oligocene ages for the third sample^18^. So, recycling of sediments through the Aveto basin is possible, although only partially.

### The Oligocene zircon roller coaster

**Figure 4:**
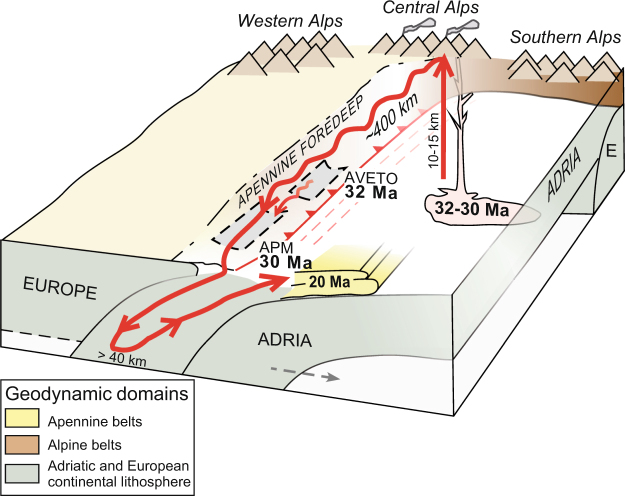
Zircon roller coaster model, not to scale. Time-transgressive (32 to 20 Ma) schematic zircon roller coaster model for the analysed samples: volcanic zircons originate in Periadriatic volcanic centres, are then translated 400 km along the Apennine foredee (similarly to what inferred for the Aveto unit^18^) after which they are subducted to 40 km depth, as indicated by high-pressure metamoprphism of the APM unit^8^, before they are subsequently rapidly exhumed by 20 Ma.

The Oligocene zircons from the APM on Elba Island record a remarkable ca. 12 Ma geodynamic history. They most likely originated from Periadriatic volcanoes of the Central Alps, when Europe collided with and was subducted underneath Adria. These volcanoes were eroded and longitudinally fed into the Apennine foredeep, where they were caught up in the Apennine subduction zone, ca. 400 km away from their volcanic origin, similar to what has been reconstructed for the Aveto basin^18^ (Fig. 4). After subduction to depth of at least 40 km, they underwent exhumation and emplacement in a nappe stack under high-pressure greenschist facies conditions by ca. 20 Ma and were subsequently rapidly brought to the surface by extensional exhumation. The rapid subduction-exhumation cycle may be explained by the collision of a microplate with a larger continent, where hinge roll back leads to extensional detachment resulting in rapid exhumation^35^. To take zircons on such a remarkable roller coaster ride thus requires two subsequent subduction zones on two different sides of the microplate (Adria): an older (Alpine) subduction underneath the microplate produces an active plate margin and the associated volcanism, followed by partly coeval (Apennine) subduction of relatively old oceanic lithosphere with an opposite subduction direction that allows for roll back, which proceeds underneath the microplate and thus yields subduction followed by rapid exhumation. In between, the erosional products of volcanoes related to the first subduction zone are transferred several hundred km by longitudinal transport along the Apennine foredeep, in analogy with the Aveto basin case^18^, before their rapid subduction and exhumation in the second subduction zone system (Fig. 4).

## Electronic supplementary material

Supplementary files
