# Supplementary material for: Alps to Apennines zircon roller coaster along the Adria microplate margin
Source: Sci Rep. 2018 Feb 9;8:2704. doi: 10.1038/s41598-018-20979-w (PMC5807382; doi:10.1038/s41598-018-20979-w)
Supplement: Supplementary file 1 — Supplementary files [file 41598_2018_20979_MOESM1_ESM.pdf]

Supplementary files

Alps to Apennines zircon roller coaster along the Adria microplate margin

Jacobs, J., Paoli, G., Rocchi, S., Ksienzyk, A.K., Sirevaag, H., Elburg, M.A.

1) Field occurrence, sample localities, petrography, geochemistry.
2) Methods: U-Pb and Lu-Hf analyses
3) U-Pb, Lu-Hf data:

   a) Cathodoluminescence

   b) U-Pb ICPMS data

   c) U-Pb SHRIMP data

   d) Lu-Hf data

4) Supporting material: Age map potential source areas, accompanying excel sheet

Supplementary File 1: Field occurrence, petrography and geochemical features of the Acquadolce phyllites and metasiltstones (APM)

Three samples were taken from the Acquadolce phyllite and metasiltstones (APM). Two samples, EJ50 and EJ67 were collected to the west of the Capo d´Arco residence in the southern part of the study area, whilst the third sample was collected along the Road to Ortano (Fig. S1a). All three samples come from silty to sandy parts of the APM.

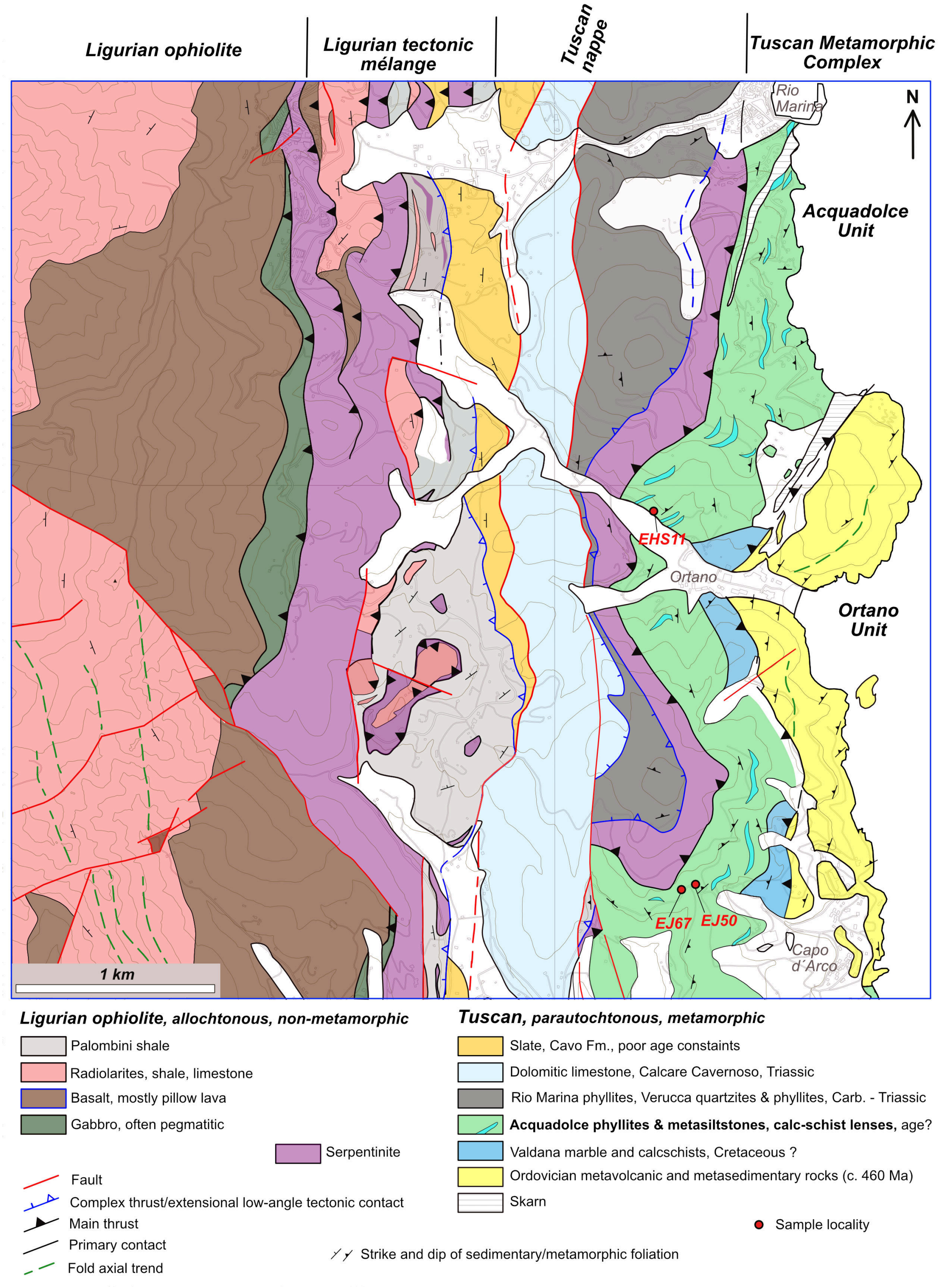

Fig. S1a: Geological map of the study area (modified after Bortolotti et al. 2001) and sample localities within the Acquadolce phyllites and metasiltstones. Map produced using Affinity Designer 1.5.5., affinity.serif.com/de/designer.

The APM are characterised by a well-developed foliation and layering on both the meso- and micro-scale, with very fine-grained layers alternating with fine-medium grained layers. The rock is highly deformed and shows common asymmetric isoclinal folding, and complex folding including sheath and oblique folds (Fig. S1b, S1c).

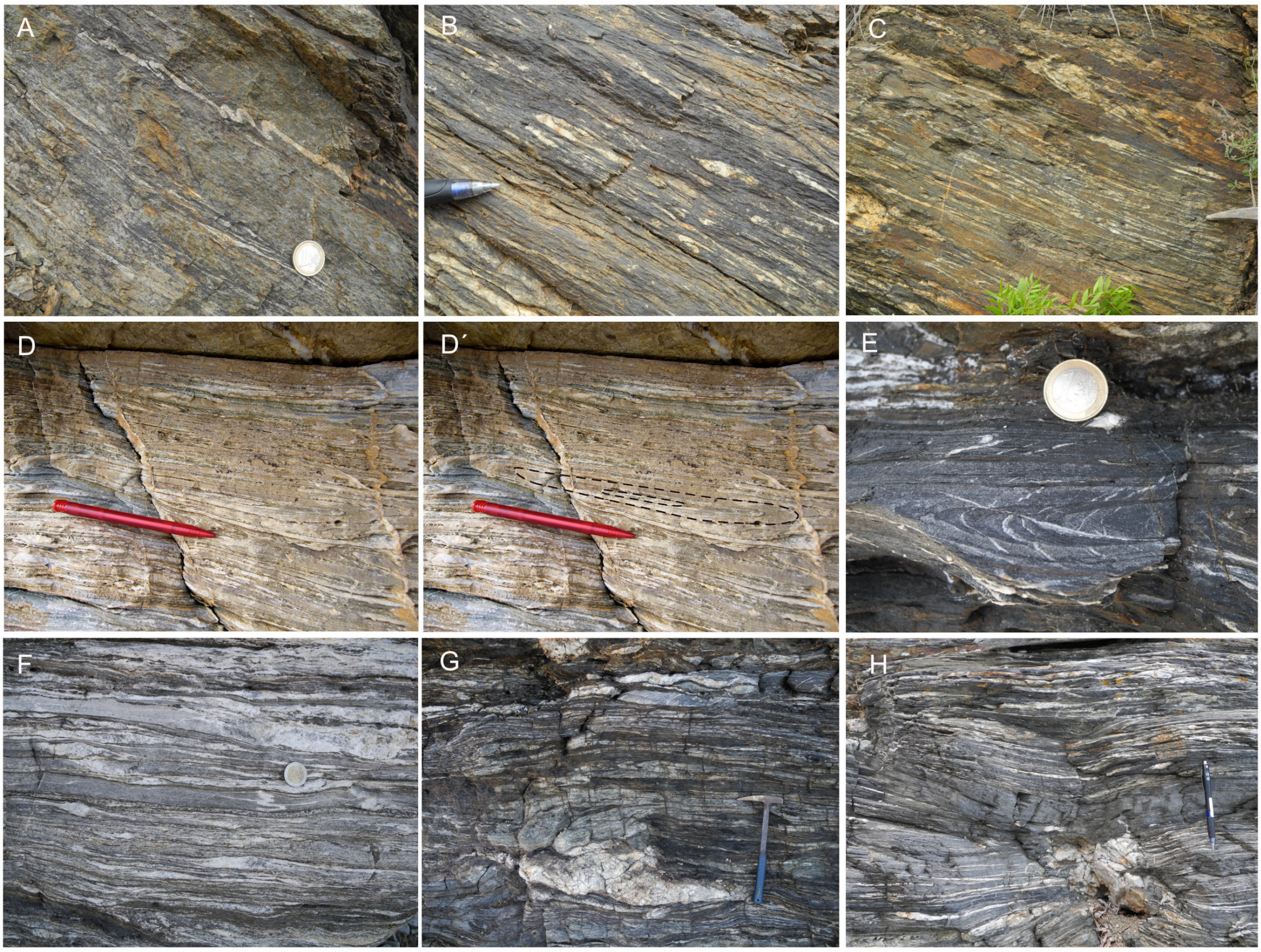

Fig. S1b: **A-C**, Isoclinal folds of alternating phyllites and metasiltstones along the road to Capo D´Arco; **D-D´**, Sheath folds in calcschists at the habour of Rio Marina; **E-H**, Complex shear folds and structural boudins in calcareous phyllites along beach outcrops in Portocillio.

The very fine-grained layers are dominated by quartz, sericite, chlorite, as well as accessory opaque minerals. In the most deformed layers, quartz aggregates are stretched to form continuous layers (Fig. S1b). The coarsest-grained layers consist of detrital K-feldspar (up to 1 mm) weakly aligned on the rock foliation in the form of elongate lenses wrapped by the fine-grained matrix, andesinic plagioclase (An_{30-35}; mineral compositions determined by SEM-EDS), and calcic pyroxene (magnesian diopside with Mg# ~0.65-0.70). Accessory minerals include titanite, with both euhedral crystals commonly partly replaced by rutile and grains affected by significant size reduction and smearing along the foliation planes. Other accessories are allanite, apatite and zircon. Frequently, K-feldspars grains are rimmed by plagioclase. These coarsest-grained layers also include large, undeformed magnesian actinolite crystals (Mg# ~0.65-0.70) poikilitically including small quartz crystals, along with albite and chlorite, representing a typical of greenschists facies assemblage after a mafic protolith (Fig. S1c).
Whole-rock chemical compositions vary depending on the layer. Two samples (EJ50G and EJ67G2, medium grained), despite their SiO_2 content typical of average upper crustal composition, have a significant content of Mg (> 3 wt%), Ca (~5.5 wt%) and Na (~2.5 wt%). On the other hand, a sample (EJ68G, fine grained) has a quite low silica content (54 wt%) coupled with

high Ca content (13 wt%). All the three samples share high contents of compatible elements like Ni, Cr and V (Table S1a-c).

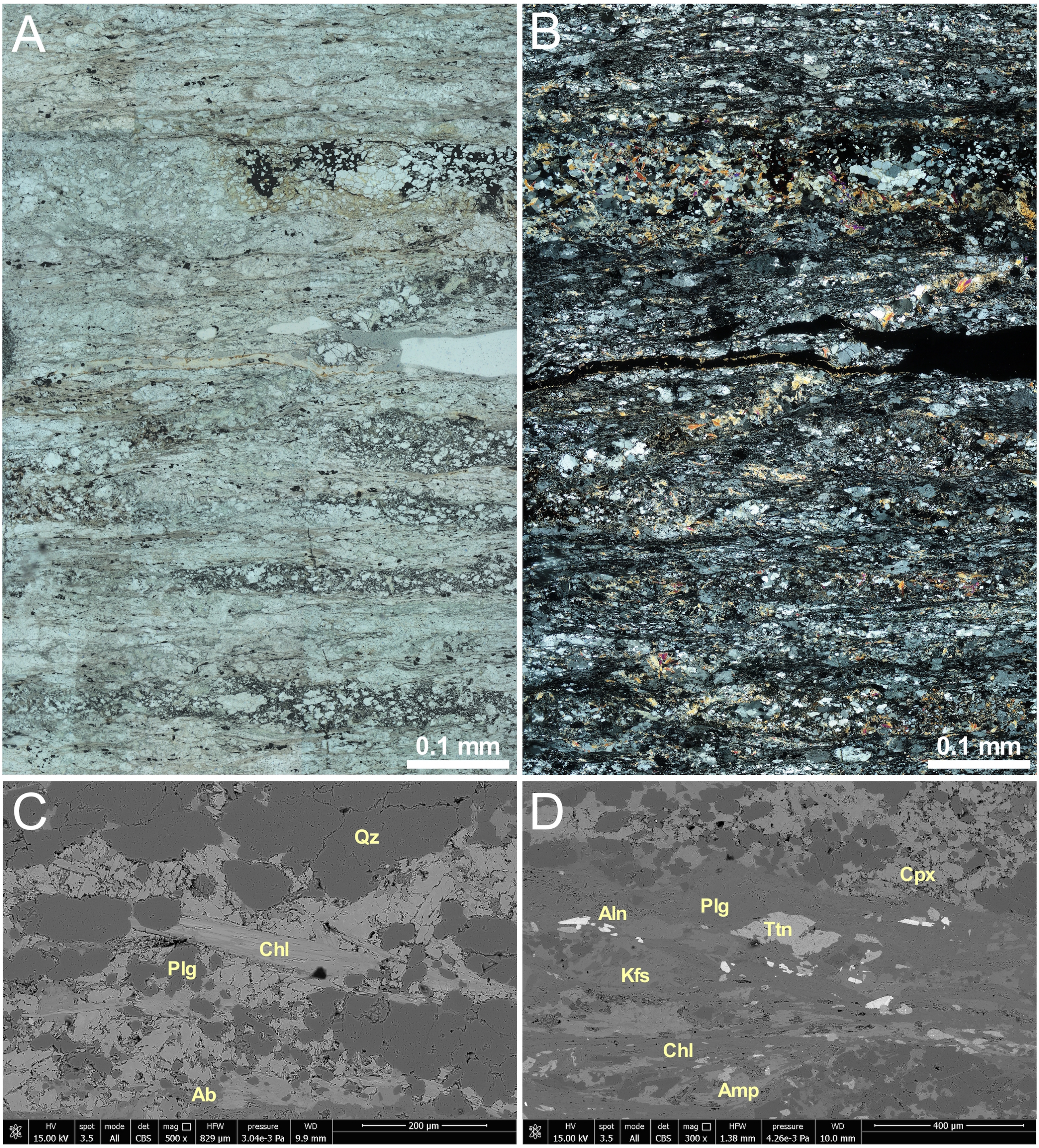

Fig. S1c: Microphotographs and FE-SEM–BSE images of Acquadolce phyllites and metasiltstone samples. **A** (plane polarized light) and **B** (crossed polars): thin sections show fine-grained layers dominated by quartz, sericite, and chlorite. The most deformed layers are characterized by quartz aggregates stretched to form continuous layers. The coarsest-grained layers consist of detrital K-feldspar (commonly rimmed by plagioclase) occasionally forming elongate lenses wrapped by fine-grained matrix, plagioclase, and calcic pyroxene. **C-D**, FE-SEM–BSE observations allow to distinguish the main accessory minerals: the most abundant accessory mineral is titanite that is characterized by euhedral crystals commonly partly replaced by rutile or grains affected by significant size reduction and smearing along the foliation planes. Other accessories are allanite, apatite and zircon. These coarsest-grained layers also include large, undeformed magnesian actinolite crystals poikilitically including small quartz crystals, along with albite and chlorite.

Table S1a. Whole-rock major and trace element composition of studied samples

	EJ 50 G	EJ 67 G2	EJ 68 G
SiO_2	67.63	68.45	54.07
TiO_2	0.56	0.52	0.78
Al_2O_3	12.73	11.81	16.10
$Fe_2O_{3\,T}$	3.71	3.89	5.92
MnO	0.16	0.20	1.14
MgO	3.38	3.44	3.02
CaO	5.72	5.33	13.13
Na_2O	2.42	2.60	0.95
K_2O	2.43	2.09	4.49
P_2O_5	0.19	0.16	0.20
Nb	8	8	16
Zr	158	140	95
Y	24	21	23
Sr	291	302	357
Rb	64	56	119
Ce	51	< d.l.	67
Ba	1085	536	824
La	32	< d.l.	< d.l.
Ni	23	60	48
Cr	140	150	71
V	71	58	108
Co	24	19	35
L.O.I.	0.99	0.27	0.57
total	99.92	98.76	100.37

major elements as wt%, trace elements as ppm; LOI: loss on ignition; detection limit

Table S1b. Pyroxene compositions – sample EJ50-EJ67

	EJ50_1	EJ50_2	EJ50_3	EJ67_1	EJ67_2
SiO_2	50.12	53.26	53.85	50.84	49.66
FeO	9.74	8.92	8.56	9.05	9.92
MgO	10.65	11.18	11.45	11.00	11.48
CaO	29.50	26.52	25.80	29.61	28.94
Wo	59.13	56.89	56.32	59.63	57.49
En	21.35	23.98	24.99	22.15	22.80
Fs	19.52	19.13	18.69	18.22	19.71
Class.	Diopside	Diopside	Diopside	Diopside	Diopside

Table S1c. Amphibole compositions for sample EJ50

crystal	1	2	3	4	5	6	7	8	9	10	11
SiO_2	50.12	55.81	55.71	56.45	54.43	53.26	53.48	57.00	53.85	54.88	57.21
Al_2O_3	< d.l.	2.26	1.81	0.17	0.27	< d.l.	0.24	1.65	0.35	4.45	2.29
FeO	9.74	13.45	12.80	12.71	15.70	8.92	14.56	10.57	8.56	13.49	13.29
MgO	10.65	15.88	15.25	15.38	13.44	11.18	13.41	17.52	11.45	15.23	12.72
CaO	29.50	12.43	14.23	14.03	13.57	26.52	13.16	13.00	25.80	11.95	14.49
Na_2O	< d.l.	0.17	0.20	0.17	0.27	< d.l.	0.24	0.27	< d.l.	< d.l.	< d.l.
IMA class.	actinol. hornbl.	actinol.	actinol.	actinol.	actinol.	actinol.	actinol.	actinol.	actinol.	actinol.	actinol.

Supplementary file 2:

Methods

Zircon grains were separated using conventional mineral separation techniques, including wet Holman-Wilfley shaking table, magnetic and heavy liquid separation. Ca. 300 zircons were handpicked, avoiding metamict grains, and mounted in epoxy. The grain mounts were then ground to section the zircons in half. After polishing, the zircons were imaged using a Zeiss Supra 55VP Scanning Electron Microscope (SEM) with cathodoluminescence detector (CL) to reveal the internal zircon structure and to guide the analysis spots (Fig. 4). One sample (EJ50) was analysed by LA-ICP-MS (unpubl. MSc thesis H. Sirevaag, 2013). The same sample as well as two additional samples (EJ60, EHS11) were thereafter analysed using SHRIMP. Thereafter, selected zircon age populations underwent Hf-characterisation.

LA-ICP-MS - U/Pb and Pb/Pb isotopic ratio analyses of zircon grains were carried out by LA–ICP-MS at the University of Bergen, using a Thermo-Finnigan Element II sector field ICP-MS system coupled with a 193 nm ArF Excimer Resonetics RESOlution M-50 LR laser. The analytical procedure is described in detail in Sirevaag et al. (2016). Laser-induced elemental fractionation and instrumental mass discrimination were corrected by normalisation to the reference zircon Plešovice (337 Ma; Sláma et al., 2008). Two standards, the 91500 zircon (1065 Ma; Wiedenbeck et al., 1995) and the GJ-1 zircon (609 Ma; Jackson et al., 2004), were analysed together with the unknowns. The 91500 zircon standard yielded an age of 1071 ± 4 Ma (n = 21, MSWD = 0.31) while the GJ-1 standard gave an age of 606 ± 3 Ma (n = 13, MSWD = 0.31).

SHRIMP U–Pb zircon analyses were carried out at the IBERSIMS facility, University of Granada. The analytical procedures largely follow Williams and Claesson (1987). The primary beam was set to an intensity of about 5 nA with a 120 µm Kohler aperture, generating 17 × 20 µm elliptical spots on the target. The secondary beam exit slit was fixed at 80 µm with a mass resolution sufficient to resolve Pb ions from molecular interferences (M/ΔM ca. 5000). All calibration procedures were performed on standards included on the same mount as the samples. Mass calibration was done on the REG zircon (ca. 2.5 Ga, very high U, Th and common lead content). Every analytical session started by measurement of the SL13 zircon, that was used as a concentration standard (238 ppm U). The TEMORA-1 zircon (416.8±1.1 Ma) was used as an isotope ratio standard and was measured between every four unknowns.

U-Pb zircon ages and data filtering: All errors are reported at the 1σ level, unless otherwise specified. For zircons younger than 1.5 Ga, $^{206}Pb/^{238}U$ ages are used and the discordance is calculated using the ratio between $^{206}Pb/^{238}U$ and $^{207}Pb/^{235}U$ age. For zircons older than 1.5 Ga, $^{207}Pb/^{206}Pb$ ages are used and the discordance is calculated using the ratio between $^{206}Pb/^{238}U$ and $^{207}Pb/^{206}Pb$ age. The datasets from both LA-ICP-MS and SHRIMP have been filtered in order to use only robust data. Analyses with 1σ errors (on the preferred age) >10% or a discordance > 10% were excluded. They are reported in Supplementary file 3A and B but are not shown on figures and are not considered for the interpretation and discussion. The age of the youngest detrital age component was determined by applying the Unmix Age algorithm from Isoplot (Ludwig, 2008) to all zircons younger than 50 Ma.

Hf isotopes on zircon were measured at the University of Johannesburg, using an ASI Resonetics 193 nm Excimer laser ablation system coupled to a Nu Plasma II multi-collector ICPMS. Ablations were done using a 50 µm diameter spot, at an ablation rate of 7 Hz and a nominal energy output of 3 mJ and 50% attenuation, which equates to a fluence of 6 J/cm^2. Further analytical details can be found in Jacobs et al. (2017). During this session, several zircon standards with varying Yb/Hf ratios were repeatedly measured giving values that are in good agreement with literature data (Tab. 1).

The measured $^{176}Hf/^{177}Hf$ ratios for the unknown were calculated to their initial value, using the measured $^{176}Lu/^{177}Hf$ ratios, the measured age, and an ^{176}Lu decay constant of $1.867*10^{-11}$ a^{-1}

(Scherer et al., 2007). The calculations of epsilon Hf were done using a present-day chondritic $^{176}Hf/^{177}Hf$ value of 0.282785 and $^{176}Lu/^{177}Hf$ of 0.0336 (Bouvier et al., 2008).

Table S2: *Comparison of measured and published values for natural zircon reference materials. Literature data from Woodhead and Hergt (2005), Heinonen et al. (2010) and Slama et al. (2008).*

sample	n	$^{176}Hf/^{177}Hf$	1s	$^{176}Lu/^{177}Hf$	1s	$^{176}Yb/^{176}Hf$	1s
Mud Tank	12	0.282514	20	0.00003	1	0.0013	3
literature		*0.282507*	*6*	*0.00004*			
Temora2	10	0.282692	24	0.00146	42	0.0527	160
literature		*0.282686*	*8*	*0.00109*			
LV11	7	0.282850	14	0.00315	9	0.1828	50
literature		*0.282837*	*14*	*0.00260*	*20*	*0.1660*	*110*
Plesovice	10	0.282487	15	0.00015	1	0.0076	5
literature		*0.282482*	*7*				

References:

Bouvier, A., Vervoort, J.D., Patchett, P.J., 2008. The Lu-Hf and Sm-Nd isotopic composition of CHUR: Constraints from unequilibrated chondrites and implications for the bulk composition of terrestrial planets. Earth and Planetary Science Letters, 273: 48-57.

Heinonen, A.P., Andersen, T., Rämö, O.T., 2010. Source constraints from the Hf isotope composition of zircon in the rapakivi granites and associated mafic rocks of southern Finland. Journal of Petrology, 51: 1687-1709.

Jackson, S. E., Pearson, N. J., Griffin, W. L. and Belousova, E. A., 2004. The application of laser ablation-inductively coupled plasma-mass spectrometry to in situ U–Pb zircon geochronology. Chemical Geology, 211: 47-69.

Jacobs, J., Opås, B., Elburg, M.A., Läufer, A., Estrada, S., Ksienzyk, A.K., Damaske, D., Hofmann, M., 2017. Cryptic sub-ice geology revealed by a U-Pb zircon study of glacial till in Dronning Maud Land, East Antarctica. Precambrian Research, 294: 1-14.

Ludwig, K. R., 2012. User's manual for Isoplot 3.75 – A geochronological toolkit for Microsoft Excel. Berkeley Geochronology Center Special Publications No. 5, 75pp.

Scherer, E.E., Munker, C., Mezger, K., 2007. The Lu-Hf systematics of meteorites: Consistent or not? Geochimica et Cosmochimica Acta, 71: A888-A888.

Slama, J., Kosler, J., Condon, D.J., Crowley, J.L., Gerdes, A., Hanchar, J.M., Horstwood, M.S.A., Morris, G.A., Nasdala, L., Norberg, N., Schaltegger, U., Schoene, B., Tubrett, M.N., Whitehouse, M.J., 2008. Plesovice zircon - A new natural reference material for U-Pb and Hf isotopic microanalysis. Chemical Geology, 249: 1-35.

Wiedenbeck, M., Allé, P., Corfu, F., Griffin, W.L., Meier, M., Oberli, F., Quadt, A.v. Roddick, J.C., Spiegel, W., 1995. Three natural zircon standards for U-Th-Pb, Lu-Hf, trace element and REE analyses. Geostandards Newsletter, 19: 1–23.

Williams, I.S. and Claesson, S. (1987). Isotopic evidence for the Precambrian provenance and Caledonian metaorphism of high grade paragneisses from the Seve Nappes, Scandinavian Caledonides. II. Ion microprobe zircon U–Th–Pb. *Contribution to Mineralogy and Petrology*, 97, 205–217.

Woodhead, J.D., Hergt, J.M., 2005. A preliminary appraisal of seven natural zircon reference materials for in situ Hf isotope determination. Geostandards and Geoanalytical Research, 29: 183-195.

Supplementary File 3a: Cathodoluminescence analyses of characteristic zircon age groups.

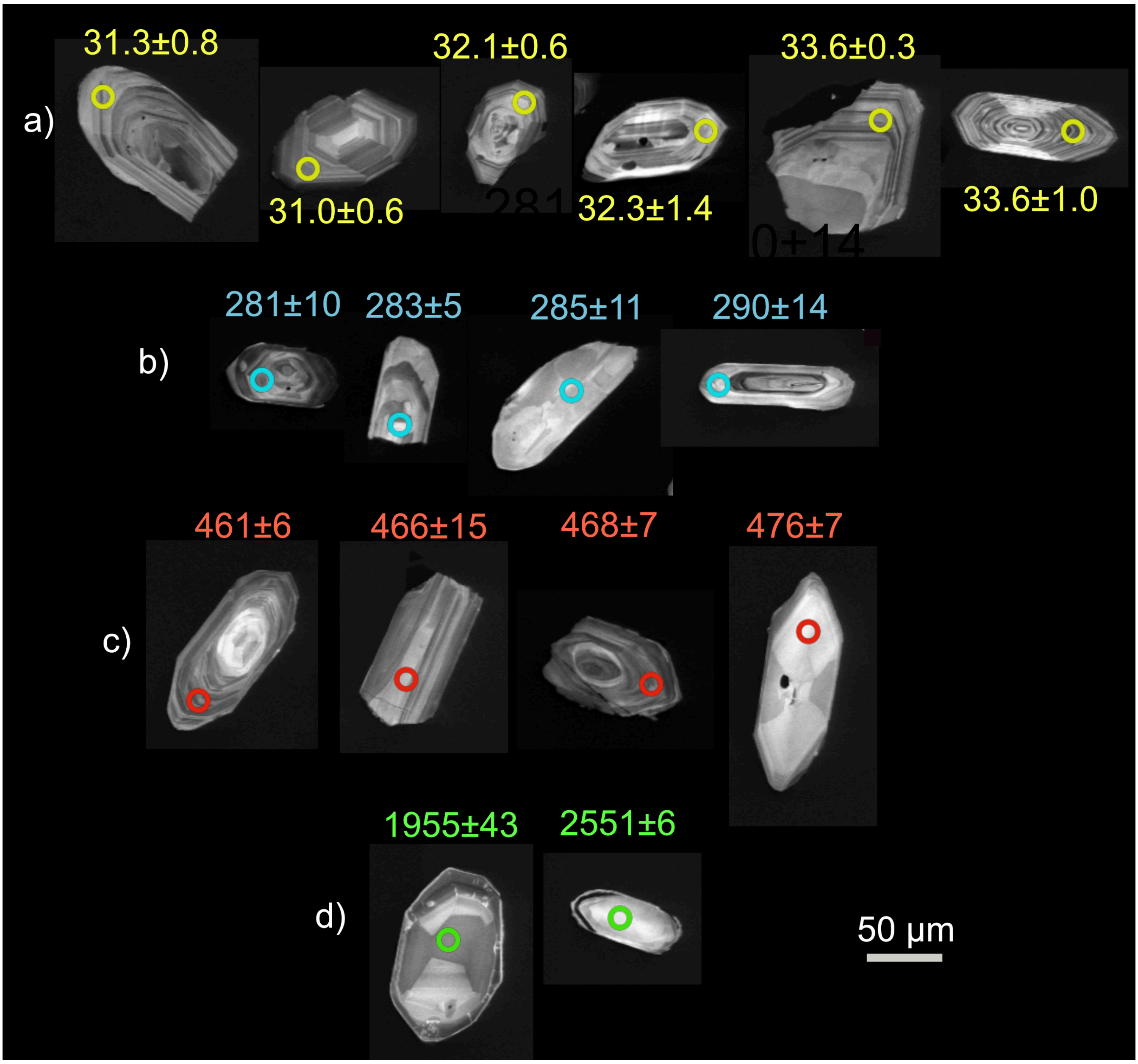

Supplementary file 3 b. Laser ablation ICP-MS U-Pb zircon analyses

		ISOTOPE RATIOS							AGES (Ma)[1]							
Analysis ID	Interpretation	$^{207}Pb/^{235}U$	± 1 σ	$^{206}Pb/^{238}U$	± 1 σ	Rho	$^{207}Pb/^{206}Pb$	± 1 σ	$^{207}Pb/^{235}U$	± 1 σ	$^{206}Pb/^{238}U$	± 1 σ	$^{207}Pb/^{206}Pb$	± 1 σ	Disc. (%)	Comment
EJ50_132	Magmatic	0.0312	0.0029	0.0046	0.0002	0.27	0.0503	0.0018	31	3	**29**	**1**	209	84	5.6	
EJ50_098	Magmatic	0.0324	0.0024	0.0047	0.0002	0.25	0.0485	0.0020	32	2	**30**	**1**	126	95	6.2	
EJ50_009	Magmatic	0.0304	0.0024	0.0048	0.0004	0.53	0.0447	0.0011	30	2	**31**	**3**	-73	62	-1.7	
EJ50_084	Magmatic	0.0317	0.0017	0.0048	0.0002	0.29	0.0472	0.0014	32	2	**31**	**1**	61	68	1.7	
EJ50_080	Magmatic	0.0297	0.0029	0.0049	0.0002	0.17	0.0474	0.0020	30	3	**31**	**1**	68	100	-5.1	
EJ50_025	Magmatic	0.0333	0.0016	0.0049	0.0002	0.35	0.0467	0.0010	33	2	**31**	**1**	31	51	5.4	
EJ50_026	Magmatic	0.0287	0.0021	0.0049	0.0002	0.30	0.0436	0.0012	29	2	**32**	**1**	-131	69	-9.7	
EJ50_125	Magmatic	0.0289	0.0038	0.0049	0.0002	0.16	0.0509	0.0018	29	4	**32**	**1**	238	80	-9.1	
EJ50_032	Magmatic	0.0298	0.0026	0.0049	0.0003	0.38	0.0432	0.0015	30	3	**32**	**2**	-154	86	-6.2	
EJ50_089	Magmatic	0.0350	0.0027	0.0050	0.0004	0.57	0.0469	0.0020	35	3	**32**	**3**	45	102	8.7	
EJ50_103	Magmatic	0.0324	0.0043	0.0050	0.0003	0.23	0.0537	0.0019	32	4	**32**	**2**	358	80	1.4	
EJ50_061	Magmatic	0.0319	0.0016	0.0050	0.0002	0.47	0.0434	0.0012	32	2	**32**	**2**	-143	69	-0.1	
EJ50_050	Magmatic	0.0311	0.0060	0.0050	0.0003	0.16	0.0531	0.0022	31	6	**32**	**2**	333	93	-2.8	
EJ50_147	Magmatic	0.0296	0.0036	0.0050	0.0002	0.17	0.0519	0.0022	30	4	**32**	**1**	279	97	-8.4	
EJ50_015	Magmatic	0.0312	0.0024	0.0050	0.0002	0.30	0.0453	0.0013	31	2	**32**	**1**	-39	71	-3.1	
EJ50_142	Magmatic	0.0318	0.0030	0.0050	0.0002	0.19	0.0507	0.0018	32	3	**32**	**1**	225	82	-1.1	
EJ50_027	Magmatic	0.0341	0.0042	0.0050	0.0004	0.32	0.0534	0.0029	34	4	**32**	**3**	346	124	5.3	
EJ50_024	Magmatic	0.0303	0.0028	0.0050	0.0003	0.28	0.0454	0.0013	30	3	**32**	**2**	-35	70	-7.1	
EJ50_008	Magmatic	0.0314	0.0035	0.0051	0.0004	0.36	0.0451	0.0017	31	3	**32**	**3**	-53	92	-3.6	
EJ50_043	Magmatic	0.0350	0.0023	0.0051	0.0002	0.29	0.0520	0.0014	35	2	**33**	**1**	286	61	6.5	
EJ50_106	Magmatic	0.0303	0.0043	0.0052	0.0003	0.17	0.0493	0.0017	30	4	**33**	**2**	161	80	-9.5	
EJ50_138	Magmatic	0.0346	0.0022	0.0052	0.0002	0.33	0.0452	0.0019	34	2	**33**	**1**	-44	100	3.5	
EJ50_118	Magmatic	0.0335	0.0067	0.0055	0.0004	0.19	0.0474	0.0020	33	7	**36**	**3**	69	99	-6.4	
EJ50_096	Magmatic	0.0391	0.0033	0.0058	0.0002	0.25	0.0499	0.0016	39	3	**37**	**2**	192	75	4.4	
EJ50_036	Magmatic	0.0405	0.0022	0.0064	0.0002	0.28	0.0479	0.0012	40	2	**41**	**1**	95	58	-1.7	
EJ50_075	Magmatic	0.0733	0.0036	0.0111	0.0004	0.33	0.0499	0.0009	72	4	**71**	**2**	193	41	0.9	
EJ50_119	Magmatic	0.1982	0.0308	0.0313	0.0025	0.26	0.0512	0.0009	184	28	**199**	**16**	248	43	-8.3	
EJ50_045	Magmatic	0.2783	0.0141	0.0382	0.0025	0.64	0.0515	0.0006	249	13	**241**	**16**	262	28	3.2	
EJ50_144	Magmatic	0.2925	0.0102	0.0400	0.0014	0.48	0.0521	0.0006	261	9	**253**	**9**	291	25	3.0	
EJ50_020	Magmatic	0.3151	0.0152	0.0432	0.0021	0.50	0.0521	0.0006	278	13	**273**	**13**	288	27	2.0	
EJ50_083	Magmatic	0.3070	0.0128	0.0439	0.0013	0.35	0.0518	0.0006	272	11	**277**	**8**	276	27	-2.0	
EJ50_035	Magmatic	0.3213	0.0150	0.0446	0.0018	0.43	0.0527	0.0006	283	13	**281**	**11**	315	26	0.5	
EJ50_077	Magmatic	0.3272	0.0123	0.0454	0.0017	0.51	0.0526	0.0006	287	11	**286**	**11**	313	26	0.3	
EJ50_140	Magmatic	0.3275	0.0171	0.0455	0.0016	0.34	0.0523	0.0009	288	15	**287**	**10**	299	37	0.3	
EJ50_031	Magmatic	0.3274	0.0192	0.0458	0.0023	0.43	0.0522	0.0007	288	17	**289**	**15**	295	29	-0.3	
EJ50_105	Uncertain	0.3175	0.0158	0.0459	0.0014	0.30	0.0504	0.0009	280	14	**289**	**9**	212	44	-3.3	
EJ50_107	Magmatic	0.3220	0.0175	0.0463	0.0014	0.28	0.0517	0.0010	283	15	**292**	**9**	273	46	-2.9	
EJ50_099	Uncertain	0.3489	0.0152	0.0469	0.0013	0.32	0.0539	0.0009	304	13	**295**	**8**	368	39	2.8	
EJ50_011	Magmatic	0.3577	0.0170	0.0493	0.0019	0.41	0.0527	0.0008	311	15	**310**	**12**	317	36	0.2	
EJ50_076	Magmatic	0.4542	0.0143	0.0577	0.0018	0.50	0.0572	0.0008	380	12	**362**	**11**	499	29	4.8	
EJ50_078	Magmatic	0.4291	0.0177	0.0596	0.0020	0.42	0.0535	0.0008	363	15	**373**	**13**	350	33	-3.0	
EJ50_067	Magmatic	0.4639	0.0563	0.0612	0.0060	0.41	0.0580	0.0009	387	47	**383**	**38**	530	34	1.0	
EJ50_060	Magmatic	0.5137	0.0327	0.0669	0.0036	0.42	0.0577	0.0007	421	27	**417**	**22**	519	26	0.8	
EJ50_042	Magmatic	0.5324	0.0270	0.0680	0.0032	0.46	0.0575	0.0008	433	22	**424**	**20**	509	30	2.2	
EJ50_097	Magmatic	0.5217	0.0256	0.0693	0.0026	0.38	0.0558	0.0006	426	21	**432**	**16**	444	26	-1.4	
EJ50_093	Magmatic	0.5321	0.0437	0.0695	0.0046	0.41	0.0567	0.0008	433	36	**433**	**29**	478	32	0.0	
EJ50_091	Magmatic	0.5506	0.0412	0.0700	0.0046	0.44	0.0579	0.0008	445	33	**436**	**29**	525	29	2.1	
EJ50_111	Magmatic	0.5345	0.0584	0.0705	0.0067	0.44	0.0550	0.0010	435	48	**439**	**42**	412	41	-1.0	
EJ50_062	Magmatic	0.5784	0.0197	0.0707	0.0032	0.66	0.0564	0.0008	463	16	**440**	**20**	469	33	5.0	
EJ50_110	Magmatic	0.5598	0.0171	0.0725	0.0015	0.35	0.0550	0.0008	451	14	**451**	**10**	414	31	0.0	
EJ50_120	Magmatic	0.5587	0.0242	0.0728	0.0020	0.32	0.0570	0.0008	451	19	**453**	**12**	491	30	-0.4	
EJ50_021	Magmatic	0.5809	0.0274	0.0730	0.0038	0.55	0.0560	0.0007	465	22	**454**	**24**	453	29	2.3	
EJ50_001	Magmatic	0.5614	0.0385	0.0734	0.0040	0.40	0.0564	0.0007	452	31	**456**	**25**	468	26	-0.9	
EJ50_065	Magmatic	0.5648	0.0453	0.0735	0.0054	0.46	0.0558	0.0010	455	36	**457**	**34**	446	38	-0.6	
EJ50_148	Magmatic	0.5325	0.0207	0.0738	0.0012	0.22	0.0567	0.0009	433	17	**459**	**8**	479	35	-5.8	
EJ50_079	Magmatic	0.5716	0.0440	0.0740	0.0057	0.50	0.0559	0.0007	459	35	**460**	**36**	448	28	-0.3	
EJ50_004	Magmatic	0.6113	0.0379	0.0743	0.0027	0.29	0.0625	0.0012	484	30	**462**	**17**	692	40	4.7	
EJ50_053	Magmatic	0.6165	0.0269	0.0746	0.0035	0.54	0.0566	0.0008	488	21	**464**	**22**	475	31	4.9	
EJ50_056	Magmatic	0.5808	0.0211	0.0746	0.0028	0.51	0.0562	0.0007	465	17	**464**	**17**	460	27	0.3	
EJ50_090	Magmatic	0.5779	0.0237	0.0748	0.0021	0.34	0.0563	0.0010	463	19	**465**	**13**	462	39	-0.4	
EJ50_018	Magmatic	0.5852	0.0258	0.0749	0.0030	0.45	0.0563	0.0007	468	21	**466**	**18**	465	27	0.5	
EJ50_130	Magmatic	0.5899	0.0228	0.0757	0.0023	0.40	0.0558	0.0007	471	18	**471**	**15**	443	27	0.1	
EJ50_128	Magmatic	0.5829	0.0471	0.0760	0.0041	0.34	0.0585	0.0010	466	38	**472**	**26**	548	36	-1.2	
EJ50_117	Magmatic	0.5677	0.0294	0.0762	0.0026	0.33	0.0571	0.0008	457	24	**474**	**16**	497	30	-3.8	
EJ50_127	Magmatic	0.6130	0.0576	0.0763	0.0066	0.46	0.0583	0.0007	485	46	**474**	**41**	541	28	2.4	
EJ50_109	Magmatic	0.6295	0.0196	0.0763	0.0013	0.28	0.0579	0.0010	496	15	**474**	**8**	526	36	4.4	
EJ50_139	Magmatic	0.5810	0.0392	0.0764	0.0039	0.38	0.0564	0.0008	465	31	**475**	**24**	470	30	-2.1	
EJ50_141	Magmatic	0.6045	0.0249	0.0773	0.0023	0.36	0.0569	0.0007	480	20	**480**	**14**	489	29	0.0	
EJ50_071	Magmatic	0.6068	0.0251	0.0778	0.0029	0.45	0.0569	0.0008	482	20	**483**	**18**	490	31	-0.3	
EJ50_017	Magmatic	0.5999	0.0321	0.0780	0.0031	0.37	0.0581	0.0008	477	26	**484**	**19**	533	29	-1.5	
EJ50_122	Magmatic	0.6266	0.0198	0.0789	0.0016	0.31	0.0580	0.0009	494	16	**490**	**10**	529	33	0.8	
EJ50_074	Magmatic	0.5899	0.0333	0.0806	0.0033	0.36	0.0566	0.0007	471	27	**500**	**20**	477	26	-6.1	
EJ50_063	Magmatic	0.6381	0.0329	0.0825	0.0035	0.41	0.0573	0.0009	501	26	**511**	**21**	504	33	-2.0	
EJ50_028	Magmatic	0.7129	0.0575	0.0849	0.0059	0.43	0.0627	0.0007	546	44	**525**	**37**	697	25	3.9	
EJ50_048	Magmatic	0.7035	0.0292	0.0860	0.0050	0.71	0.0573	0.0008	541	22	**532**	**31**	504	32	1.6	
EJ50_126	Magmatic	0.7433	0.0361	0.0898	0.0031	0.36	0.0607	0.0009	564	27	**554**	**19**	627	31	1.8	
EJ50_044	Uncertain	0.7005	0.0509	0.0905	0.0050	0.38	0.0584	0.0008	539	39	**559**	**31**	544	32	-3.6	
EJ50_059	Magmatic	0.7702	0.0362	0.0915	0.0045	0.53	0.0601	0.0007	580	27	**564**	**28**	607	24	2.7	
EJ50_049	Magmatic	0.7114	0.0536	0.0920	0.0050	0.36	0.0596	0.0009	546	41	**567**	**31**	589	32	-4.0	
EJ50_052	Magmatic	0.7908	0.0298	0.0931	0.0039	0.56	0.0602	0.0006	592	22	**574**	**24**	611	22	3.0	
EJ50_029	Magmatic	0.7442	0.0444	0.0946	0.0045	0.40	0.0592	0.0007	565	34	**583**	**28**	574	26	-3.2	
EJ50_073	Magmatic	0.7905	0.0592	0.0960	0.0060	0.42	0.0614	0.0009	591	44	**591**	**37**	654	30	0.0	
EJ50_116	Magmatic	0.8097	0.0370	0.0978	0.0041	0.46	0.0598	0.0008	602	28	**601**	**25**	595	27	0.2	
EJ50_101	Magmatic	0.7975	0.0372	0.0998	0.0028	0.30	0.0604	0.0008	595	28	**613**	**17**	618	28	-3.0	
EJ50_135	Uncertain	0.8160	0.0322	0.1001	0.0025	0.32	0.0606	0.0008	606	24	**615**	**15**	627	27	-1.5	
EJ50_023	Uncertain	0.8825	0.0335	0.1002	0.0040	0.53	0.0614	0.0007	642	24	**616**	**25**	655	25	4.1	
EJ50_022	Magmatic	0.8221	0.0330	0.1003	0.0034	0.42	0.0595	0.0007	609	24	**616**	**21**	587	27	-1.1	
EJ50_002	Magmatic	0.8293	0.0411	0.1008	0.0035	0.35	0.0609	0.0010	613	30	**619**	**22**	635	35	-1.0	
EJ50_030	Uncertain	0.9352	0.0956	0.1011	0.0075	0.36	0.0684	0.0012	670	69	**621**	**46**	881	37	7.4	
EJ50_085	Magmatic	0.8319	0.0435	0.1012	0.0035	0.33	0.0612	0.0008	615	32	**622**	**21**	647	29	-1.1	
EJ50_041	Magmatic	0.8080	0.0555	0.1021	0.0036	0.26	0.0600	0.0007	601	41	**627**	**22**	602	26	-4.3	
EJ50_114	Magmatic	0.9162	0.0358	0.1041	0.0033	0.40	0.0623	0.0010	660	26	**638**	**20**	684	33	3.3	
EJ50_013	Magmatic	0.9785	0.0372	0.1132	0.0038	0.45	0.0617	0.0008	693	26	**692**	**23**	663	27	0.2	
EJ50_137	Uncertain	1.0139	0.0924	0.1135	0.0076	0.37	0.0686	0.0010	711	65	**693**	**46**	885	29	2.5	
EJ50_055	Magmatic	0.9956	0.0392	0.1141	0.0042	0.46	0.0628	0.0009	702	28	**697**	**25**	701	30	0.7	
EJ50_034	Magmatic	0.9693	0.0488	0.1145	0.0044	0.38	0.0634	0.0009	688	35	**699**	**27**	721	31	-1.5	
EJ50_007	Magmatic	1.0936	0.0782	0.1272	0.0064	0.35	0.0648	0.0008	750	54	**772**	**39**	767	27	-2.9	
EJ50_037	Magmatic	1.1614	0.0472	0.1274	0.0047	0.45	0.0655	0.0008	783	32	**773**	**28**	789	25	1.2	
EJ50_149	Uncertain	1.1538	0.0528	0.1296	0.0051	0.43	0.0647	0.0009	779	36	**785**	**31**	765	28	-0.8	
EJ50_086	Magmatic	1.2658	0.0490	0.1320	0.0037	0.36	0.0655	0.0014	830	32	**799**	**22**	789	46	3.8	
EJ50_082	Magmatic	1.2025	0.0606	0.1364	0.0043	0.31	0.0663	0.0010	802	40	**824**	**26**	816	31	-2.8	
EJ50_134	Magmatic	1.2828	0.0512	0.1404	0.0044	0.39	0.0670	0.0007	838	33	**847**	**27**	838	22	-1.1	
EJ50_064	Magmatic	1.2863	0.0496	0.1411	0.0051	0.47	0.0662	0.0009	840	32	**851**	**31**	813	28	-1.4	
EJ50_005	Magmatic	1.4262	0.0633	0.1506	0.0059	0.44	0.0677	0.0010	900	40	**904**	**35**	861	32	-0.5	
EJ50_108	Inheritance	1.5749	0.0601	0.1564	0.0043	0.36	0.0724	0.0010	960	37	**936**	**26**	998	28	2.5	
EJ50_143	Magmatic	1.7431	0.0658	0.1666	0.0047	0.37	0.0748	0.0010	1025	39	**994**	**28**	1063	28	3.0	
EJ50_058	Uncertain	5.4918	0.1489	0.3353	0.0116	0.64	0.1159	0.0012	1899	52	1864	65	**1894**	**19**	1.6	
EJ50_033	Magmatic	5.6384	0.2120	0.3410	0.0139	0.54	0.1172	0.0015	1922	72	1891	77	**1914**	**22**	1.2	
EJ50_057	Uncertain	5.7141	0.2354	0.3490	0.0157	0.55	0.1179	0.0010	1934	80	1930	87	**1925**	**14**	-0.3	
EJ50_051	Magmatic	7.0814	0.2925	0.3827	0.0207	0.65	0.1298	0.0017	2122	88	2089	113	**2095**	**12**	0.3	
EJ50_069	Magmatic	11.3609	0.6392	0.4654	0.0262	0.50	0.1779	0.0018	2553	144	2463	139	**2634**	**16**	6.5	
EJ50_038	Uncertain	13.8999	0.5160	0.5209	0.0200	0.52	0.1870	0.0019	2743	102	2703	104	**2716**	**17**	0.5	

Supplementary file 3 b_continued. Laser ablation ICP-MS U-Pb zircon analyses

		ISOTOPE RATIOS							AGES (Ma)[1]							
Analysis ID	Interpretation	$^{207}Pb/^{235}U$	± 1 σ	$^{206}Pb/^{238}U$	± 1 σ	Rho	$^{207}Pb/^{206}Pb$	± 1 σ	$^{207}Pb/^{235}U$	± 1 σ	$^{206}Pb/^{238}U$	± 1 σ	$^{207}Pb/^{206}Pb$	± 1 σ	Disc. (%)	Comment
**Excluded analyses (discordant or errors > 10 %)**																
EJ50_068	Magmatic	0.0425	0.0034	0.0045	0.0007	0.99	0.0586	0.0028	42	3	**29**	**5**	551	105	32.2	Discordant, error > 10 %
EJ50_104	Magmatic	0.0220	0.0034	0.0046	0.0003	0.18	0.0415	0.0020	22	3	**30**	**2**	-260	120	-35.4	Discordant
EJ50_124	Magmatic	0.0334	0.0053	0.0047	0.0003	0.23	0.0587	0.0025	33	5	**30**	**2**	556	93	10.2	Discordant
EJ50_087	Magmatic	0.0371	0.0024	0.0047	0.0002	0.38	0.0532	0.0018	37	2	**31**	**1**	338	79	17.5	Discordant
EJ50_003	Magmatic	0.0389	0.0039	0.0048	0.0004	0.44	0.0496	0.0017	39	4	**31**	**3**	177	78	20.6	Discordant
EJ50_039	Magmatic	0.0352	0.0037	0.0048	0.0003	0.27	0.0555	0.0020	35	4	**31**	**2**	431	81	12.5	Discordant
EJ50_129	Magmatic	0.0245	0.0097	0.0048	0.0005	0.13	0.0474	0.0031	25	10	**31**	**3**	70	155	-26.8	Discordant, error > 10 %
EJ50_150	Magmatic	0.0358	0.0021	0.0049	0.0001	0.23	0.0515	0.0019	36	2	**31**	**1**	264	84	12.2	Discordant
EJ50_010	Magmatic	0.0381	0.0025	0.0049	0.0003	0.42	0.0517	0.0019	38	2	**32**	**2**	273	83	16.8	Discordant
EJ50_121	Magmatic	0.0413	0.0034	0.0050	0.0002	0.18	0.0640	0.0023	41	3	**32**	**1**	743	77	21.1	Discordant
EJ50_047	Magmatic	0.0440	0.0031	0.0050	0.0003	0.46	0.0579	0.0018	44	3	**32**	**2**	524	69	25.8	Discordant
EJ50_092	Magmatic	0.0280	0.0039	0.0051	0.0003	0.20	0.0450	0.0018	28	4	**33**	**2**	-56	96	-16.3	Discordant
EJ50_066	Magmatic	0.0369	0.0028	0.0051	0.0002	0.31	0.0509	0.0019	37	3	**33**	**2**	235	84	10.8	Discordant
EJ50_100	Magmatic	0.0702	0.0081	0.0052	0.0003	0.28	0.0989	0.0062	69	8	**33**	**2**	1603	118	51.8	Discordant
EJ50_006	Magmatic	0.0423	0.0029	0.0052	0.0002	0.28	0.0576	0.0021	42	3	**33**	**1**	514	80	21.0	Discordant
EJ50_094	Magmatic	0.0628	0.0059	0.0052	0.0001	0.14	0.0761	0.0049	62	6	**33**	**1**	1098	129	46.0	Discordant
EJ50_081	Magmatic	0.0302	0.0045	0.0055	0.0002	0.10	0.0548	0.0026	30	4	**35**	**1**	405	107	-17.0	Discordant
EJ50_123	Magmatic	0.0707	0.0069	0.0056	0.0001	0.14	0.0726	0.0040	69	7	**36**	**1**	1002	112	47.9	Discordant
EJ50_012	Magmatic	0.0405	0.0032	0.0056	0.0003	0.37	0.0468	0.0020	40	3	**36**	**2**	41	104	10.2	Discordant
EJ50_070	Magmatic	0.0609	0.0080	0.0060	0.0002	0.14	0.0665	0.0026	60	8	**39**	**1**	821	83	35.7	Discordant
EJ50_046	Uncertain	0.0419	0.0251	0.0061	0.0008	0.11	0.0593	0.0022	42	25	**39**	**5**	577	80	5.9	Error > 10 %
EJ50_133	Magmatic	0.0345	0.0046	0.0063	0.0002	0.13	0.0521	0.0018	34	5	**41**	**1**	289	80	-18.3	Discordant
EJ50_145	Uncertain	0.0933	0.0089	0.0103	0.0005	0.23	0.0649	0.0037	91	9	**66**	**3**	773	121	27.1	Discordant
EJ50_102	Magmatic	0.1792	0.0193	0.0241	0.0028	0.54	0.0797	0.0048	167	18	**154**	**18**	1190	119	8.1	Error > 10 %
EJ50_136	Magmatic	0.2384	0.1175	0.0348	0.0051	0.15	0.0724	0.0027	217	107	**220**	**32**	998	75	-1.5	Error > 10 %
EJ50_131	Magmatic	0.2991	0.0720	0.0434	0.0078	0.37	0.0521	0.0012	266	64	**274**	**49**	291	51	-3.2	Error > 10 %
EJ50_146	Magmatic	0.5028	0.1684	0.0625	0.0178	0.43	0.0595	0.0017	414	139	**391**	**111**	586	64	5.5	Error > 10 %
EJ50_095	Uncertain	0.4954	0.1142	0.0675	0.0108	0.35	0.0561	0.0011	409	94	**421**	**67**	456	45	-3.0	Error > 10 %
EJ50_115	Magmatic	0.9033	0.0472	0.0680	0.0031	0.44	0.0820	0.0036	654	34	**424**	**20**	1247	86	35.1	Discordant
EJ50_072	Magmatic	0.5805	0.1266	0.0718	0.0079	0.25	0.0619	0.0022	465	101	**447**	**49**	670	77	3.8	Error > 10 %
EJ50_014	Magmatic	0.6030	0.2374	0.0762	0.0181	0.30	0.0646	0.0018	479	189	**473**	**112**	762	59	1.2	Error > 10 %
EJ50_113	Magmatic	0.8200	0.0486	0.0826	0.0037	0.38	0.0618	0.0019	608	36	**512**	**23**	667	64	15.8	Discordant
EJ50_088	Magmatic	0.8838	0.1680	0.1244	0.0037	0.08	0.0641	0.0007	643	122	**756**	**22**	745	25	-17.5	Discordant
EJ50_019	Uncertain	2.5903	0.7550	0.2030	0.0319	0.27	0.1041	0.0021	1298	378	**1191**	**188**	1698	37	8.2	Error > 10 %
EJ50_016	Magmatic	5.0153	0.3335	0.2889	0.0185	0.48	0.1241	0.0012	1822	121	1636	105	**2016**	**18**	18.9	Discordant
EJ50_112	Magmatic	8.8776	1.1831	0.3846	0.0608	0.59	0.1629	0.0021	2326	310	2098	331	**2486**	**21**	15.6	Discordant

Errors are at 1 σ level. Plešovice (337 Ma; Sláma et al., 2008) was used as the primary standard to correct for laser-induced elemental fractionation and instrumental mass discrimination.

Standards 91500 (1065 Ma; Wiedenbeck et al., 1995) and GJ-1 (609 Ma; Jackson et al., 2004) were used as control samples and gave ages of 1071 ± 4 Ma (n = 21) and 606 ± 3 Ma (n = 13), respectively.

For zircons older than 1.5 Ga, the 207Pb/206Pb age is preferred, and the discordance is calculated Disc. (%) = (206Pb/238U age / 207Pb/206Pb age) x 100.

[1]The preferred ages are in bold numbers (and highlighted in green). For zircons younger than 1.5 Ga, the $^{206}Pb/^{238}U$ age is preferred, and the discordance is calculated Disc. (%) = ($^{206}Pb/^{238}U$ age / $^{207}Pb/^{235}$ U age) x 100.

Supplementary file 3B: SHRIMP U-Pb zircon analyses of samples EJ50, JF67 and EHS ordered after their preferred age

								ISOTOPE RATIOS											AGES (Ma)[2]							
Analysis ID	Interpretation	U (ppm)	Th (ppm)	^{206}Pb (ppm)	f206_4 (%)[1]	f206_8 (%)[1]	Th/U	$^{207}Pb/^{235}U$	± 1 σ	$^{206}Pb/^{238}U$	± 1 σ	Rho	$^{207}Pb/^{206}Pb$	± 1 σ	$^{204}Pb/^{206}Pb$	± 1 σ	$^{208}Pb/^{232}Th$	± 1 σ	$^{207}Pb/^{235}U$	± 1 σ	$^{206}Pb/^{238}U$	± 1 σ	$^{207}Pb/^{206}Pb$	± 1 σ	Disc. (%)	Comment
EJ50-19.1	Magmatic	751	416	3.1	3.07	0.34	0.57	0.0325	0.0014	0.0048	0.0001	0.44	0.0490	0.0017	0.0016	0.0006	0.0016	0.0000	33	1	**31**	**1**	148	77	4.9	
EJ67-38.1	Magmatic	1054	607	4.4	0.92	0.67	0.59	0.0321	0.0008	0.0048	0.0001	0.57	0.0484	0.0007	0.0005	0.0002	0.0016	0.0000	32	1	**31**	**1**	119	34	3.4	
EJ67-25.1	Magmatic	581	121	2.5	0.73	0.29	0.21	0.0328	0.0013	0.0049	0.0001	0.47	0.0488	0.0015	0.0004	0.0001	0.0017	0.0001	33	1	**31**	**1**	138	72	4.6	
EJ50-36.1	Magmatic	824	530	3.6	0.30	0.12	0.66	0.0323	0.0007	0.0050	0.0001	0.55	0.0470	0.0007	0.0002	0.0001	0.0016	0.0000	32	1	**32**	**1**	0	0	0.6	
EHS-26.1	Magmatic	635	598	2.8	2.54	0.30	0.97	0.0337	0.0018	0.0050	0.0002	0.59	0.0487	0.0015	0.0014	0.0004	0.0016	0.0001	34	2	**32**	**1**	134	70	4.2	
EHS-05.1	Magmatic	1086	313	4.8	1.74	0.42	0.30	0.0346	0.0018	0.0051	0.0003	0.68	0.0496	0.0008	0.0009	0.0004	0.0018	0.0001	35	2	**33**	**2**	178	38	5.8	
EJ67-37.1	Magmatic	1106	132	5.0	0.91	0.19	0.12	0.0339	0.0004	0.0052	0.0001	0.61	0.0470	0.0003	0.0005	0.0002	0.0018	0.0000	34	0	**34**	**0**	0	0	0.6	
EJ67-07.1	Magmatic	884	807	4.0	-0.19	1.25	0.94	0.0339	0.0011	0.0052	0.0002	0.65	0.0470	0.0007	-0.0001	0.0003	0.0018	0.0001	34	1	**34**	**1**	0	0	0.6	
EJ50-27.1	Magmatic	693	299	3.1	1.57	0.13	0.44	0.0353	0.0008	0.0052	0.0001	0.58	0.0488	0.0006	0.0008	0.0002	0.0017	0.0000	35	1	**34**	**1**	138	30	4.3	
EHS-12.2	Magmatic	648	286	3.1	2.61	0.39	0.45	0.0389	0.0020	0.0055	0.0003	0.66	0.0515	0.0011	0.0014	0.0005	0.0018	0.0001	39	2	**35**	**2**	262	47	9.0	
EHS-11.2	Magmatic	749	345	3.6	0.49	0.20	0.47	0.0371	0.0014	0.0055	0.0002	0.55	0.0490	0.0012	0.0003	0.0002	0.0018	0.0001	37	1	**35**	**1**	148	56	4.6	
EHS-34.1	Magmatic	636	256	3.1	1.02	0.63	0.41	0.0370	0.0013	0.0056	0.0001	0.40	0.0481	0.0014	0.0005	0.0002	0.0019	0.0001	37	1	**36**	**1**	104	70	2.7	
EHS-03.1	Magmatic	638	269	3.2	1.41	0.29	0.43	0.0381	0.0014	0.0058	0.0001	0.39	0.0472	0.0015	0.0008	0.0005	0.0019	0.0000	38	1	**38**	**1**	0	133	1.1	
EHS-14.1	Magmatic	956	389	4.9	1.15	-0.34	0.42	0.0398	0.0018	0.0059	0.0002	0.61	0.0487	0.0011	0.0006	0.0002	0.0018	0.0001	40	2	**38**	**1**	134	54	3.8	
EHS-33.1	Magmatic	777	541	4.1	1.54	0.21	0.71	0.0411	0.0017	0.0061	0.0002	0.59	0.0487	0.0011	0.0008	0.0003	0.0020	0.0001	41	2	**39**	**1**	135	54	3.9	
EJ67-24.1	Magmatic	836	312	4.6	0.40	0.05	0.38	0.0429	0.0009	0.0064	0.0001	0.43	0.0486	0.0008	0.0002	0.0001	0.0021	0.0000	43	1	**41**	**1**	129	41	3.5	
EJ50-14.1	Magmatic	661	301	21.0	0.72	0.56	0.47	0.2693	0.0070	0.0366	0.0008	0.60	0.0533	0.0008	0.0004	0.0002	0.0124	0.0003	242	6	**232**	**5**	342	33	4.2	
EJ67-15.1	Recrsytallised	228	72	8.0	0.31	0.38	0.32	0.2794	0.0089	0.0409	0.0013	0.70	0.0496	0.0004	0.0002	0.0001	0.0138	0.0005	250	7	**258**	**8**	177	19	-3.2	
EJ67-08.1	Uncertain	215	145	7.8	0.34	-0.22	0.69	0.2975	0.0130	0.0421	0.0017	0.68	0.0512	0.0007	0.0002	0.0001	0.0130	0.0005	264	10	**266**	**11**	252	31	-0.6	
EHS-22.1	Magmatic	123	77	4.7	-0.21	0.02	0.64	0.3118	0.0068	0.0438	0.0005	0.38	0.0516	0.0010	-0.0001	0.0002	0.0138	0.0002	276	5	**276**	**3**	270	42	-0.3	
EJ67-18.1	Recrsytallised	344	125	13.0	0.37	-0.17	0.37	0.3079	0.0073	0.0438	0.0010	0.67	0.0509	0.0005	0.0002	0.0001	0.0134	0.0003	273	6	**277**	**6**	239	20	-1.4	
EHS-10.1	Magmatic	625	247	24.1	0.02	0.02	0.41	0.3150	0.0116	0.0445	0.0016	0.70	0.0513	0.0004	0.0000	0.0000	0.0140	0.0005	278	9	**281**	**10**	257	20	-0.9	
EHS-17.1	Recrsytallised	257	182	10.0	0.64	0.66	0.73	0.3219	0.0066	0.0449	0.0008	0.61	0.0520	0.0006	0.0003	0.0001	0.0149	0.0003	283	5	**283**	**5**	285	24	0.0	
EHS-15.1	Recrsytallised	126	69	4.9	0.42	-0.05	0.56	0.3308	0.0144	0.0452	0.0017	0.63	0.0531	0.0011	0.0002	0.0003	0.0141	0.0006	290	11	**285**	**11**	332	47	1.8	
EJ67-05.1	Magmatic	126	105	5.0	1.60	0.23	0.86	0.3258	0.0117	0.0456	0.0016	0.68	0.0519	0.0006	0.0009	0.0002	0.0145	0.0005	286	9	**287**	**10**	280	26	-0.3	
EJ50-37.1	Magmatic	402	111	16.0	0.18	0.03	0.28	0.3353	0.0168	0.0461	0.0023	0.71	0.0528	0.0004	0.0001	0.0000	0.0146	0.0007	294	13	**290**	**14**	320	15	1.1	
EJ67-31.1	Magmatic	370	144	14.8	0.12	0.11	0.40	0.3295	0.0094	0.0462	0.0013	0.69	0.0517	0.0004	0.0001	0.0001	0.0148	0.0004	289	7	**291**	**8**	274	19	-0.7	
EJ50-04.1	Magmatic	309	164	12.4	0.51	0.24	0.54	0.3347	0.0061	0.0464	0.0008	0.69	0.0524	0.0003	0.0003	0.0001	0.0150	0.0003	293	5	**292**	**5**	301	12	0.3	
EHS-07.1	Magmatic	380	222	15.6	0.09	0.03	0.60	0.3329	0.0077	0.0474	0.0009	0.61	0.0509	0.0006	0.0000	0.0001	0.0149	0.0003	292	6	**299**	**6**	237	28	-2.4	
EJ67-41.1	Magmatic	184	78	7.6	0.32	-0.01	0.43	0.3472	0.0061	0.0476	0.0008	0.69	0.0529	0.0003	0.0002	0.0001	0.0149	0.0005	303	5	**300**	**5**	326	12	1.0	
EHS-16.1	Magmatic	1452	43	60.2	0.28	0.12	0.03	0.3404	0.0059	0.0479	0.0008	0.70	0.0515	0.0002	0.0002	0.0000	0.0191	0.0005	298	5	**302**	**5**	264	10	-1.4	
EJ67-17.1	Magmatic	470	126	19.7	0.13	-0.12	0.27	0.3445	0.0100	0.0483	0.0014	0.70	0.0518	0.0003	0.0001	0.0000	0.0147	0.0005	301	8	**304**	**9**	275	14	-1.1	
EJ50-11.1	Magmatic	455	231	19.5	-0.08	-0.05	0.52	0.3534	0.0111	0.0496	0.0015	0.68	0.0516	0.0005	0.0000	0.0000	0.0155	0.0005	307	8	**312**	**9**	269	23	-1.7	
EHS-31.1	Magmatic	650	905	29.2	0.23	-0.61	1.43	0.3825	0.0043	0.0519	0.0004	0.52	0.0534	0.0004	0.0001	0.0000	0.0159	0.0002	329	3	**327**	**3**	346	18	0.7	
EJ50-21.1	Recrsytallised	310	115	14.2	0.28	0.00	0.38	0.3879	0.0141	0.0531	0.0019	0.71	0.0530	0.0004	0.0001	0.0000	0.0166	0.0006	333	10	**333**	**12**	330	15	-0.2	
EHS-32.1	Metamorphic	197	15	9.2	0.05	0.14	0.08	0.4110	0.0071	0.0541	0.0008	0.59	0.0551	0.0005	0.0000	0.0000	0.0190	0.0008	350	5	**340**	**5**	415	22	2.8	
EHS-04.1	Magmatic	301	132	14.5	0.40	0.18	0.45	0.4054	0.0124	0.0555	0.0017	0.71	0.0529	0.0003	0.0002	0.0001	0.0178	0.0006	346	9	**348**	**10**	326	12	-0.8	
EJ50-18.1	Magmatic	1194	1023	60.2	0.11	-0.23	0.88	0.4253	0.0091	0.0583	0.0012	0.70	0.0529	0.0003	0.0001	0.0000	0.0180	0.0004	360	7	**365**	**7**	325	11	-1.5	
EJ67-11.1	Magmatic	604	463	30.5	0.14	0.29	0.79	0.4259	0.0129	0.0583	0.0018	0.72	0.0529	0.0002	0.0001	0.0000	0.0186	0.0006	360	9	**366**	**11**	327	7	-1.4	
EHS-28.1	Metamorphic	665	15	34.7	0.14	0.06	0.02	0.4463	0.0110	0.0603	0.0014	0.68	0.0537	0.0004	0.0001	0.0000	0.0223	0.0008	375	8	**377**	**9**	359	17	-0.7	
EHS-38.1	Recrsytallised	315	6	17.7	0.01	0.04	0.02	0.5012	0.0026	0.0650	0.0003	0.72	0.0560	0.0000	0.0000	0.0000	0.0226	0.0001	413	2	**406**	**2**	451	0	1.6	
EJ50-24.1	Magmatic	512	8	30.9	0.63	0.44	0.02	0.5691	0.0078	0.0697	0.0008	0.58	0.0592	0.0005	0.0003	0.0001	0.0617	0.0035	457	5	**435**	**5**	574	17	5.0	
EHS-27.1	Magmatic	383	97	23.6	0.12	0.30	0.26	0.5479	0.0144	0.0712	0.0017	0.67	0.0558	0.0006	0.0001	0.0000	0.0238	0.0008	444	10	**443**	**10**	446	22	0.1	
EJ50-23.1	Recrsytallised	183	26	11.4	0.45	0.05	0.15	0.5616	0.0271	0.0721	0.0035	0.71	0.0565	0.0004	0.0002	0.0001	0.0230	0.0011	453	18	**449**	**21**	472	14	0.8	
EHS-01.1	Magmatic	277	53	17.5	0.78	0.65	0.20	0.6149	0.0160	0.0730	0.0018	0.70	0.0611	0.0004	0.0004	0.0001	0.0277	0.0007	487	10	**454**	**11**	642	13	6.6	
EHS-30.1	Magmatic	269	46	17.0	0.15	0.02	0.17	0.5561	0.0149	0.0731	0.0019	0.71	0.0552	0.0002	0.0001	0.0000	0.0229	0.0008	449	10	**455**	**12**	420	9	-1.3	
EHS-37.1	Magmatic	445	99	28.3	-0.18	0.37	0.23	0.5753	0.0066	0.0734	0.0008	0.72	0.0568	0.0000	-0.0001	0.0000	0.0253	0.0003	461	4	**457**	**5**	485	0	1.0	
EJ67-30.1	Magmatic	574	47	36.6	0.16	0.00	0.08	0.5725	0.0177	0.0736	0.0022	0.69	0.0564	0.0005	0.0001	0.0000	0.0230	0.0009	460	12	**458**	**13**	470	19	0.5	
EHS-39.1	Magmatic	449	53	28.6	0.26	0.13	0.12	0.5892	0.0027	0.0736	0.0003	0.72	0.0580	0.0000	0.0001	0.0000	0.0245	0.0001	470	2	**458**	**2**	531	0	2.6	
EJ67-29.1	Magmatic	793	66	50.9	0.09	-0.02	0.09	0.5678	0.0086	0.0741	0.0010	0.67	0.0556	0.0003	0.0000	0.0000	0.0226	0.0004	457	6	**461**	**6**	435	13	-0.9	
EJ67-12.1	Magmatic	299	68	19.4	-0.01	-0.12	0.23	0.5819	0.0193	0.0749	0.0024	0.70	0.0564	0.0005	0.0000	0.0001	0.0225	0.0008	466	13	**466**	**15**	467	18	0.0	
EJ50-41.1	Magmatic	197	105	12.8	0.16	-0.10	0.55	0.5743	0.0051	0.0749	0.0007	0.72	0.0556	0.0000	0.0001	0.0000	0.0231	0.0002	461	3	**466**	**4**	437	0	-1.0	
EHS-23.1	Magmatic	324	52	21.1	0.31	0.21	0.16	0.5799	0.0101	0.0753	0.0012	0.65	0.0559	0.0004	0.0002	0.0000	0.0254	0.0005	464	7	**468**	**7**	448	16	-0.7	
EJ50-12.1	Recrsytallised	440	53	29.1	0.45	0.23	0.12	0.5973	0.0113	0.0765	0.0014	0.71	0.0567	0.0002	0.0002	0.0001	0.0267	0.0006	476	7	**475**	**9**	479	7	0.1	
EJ50-01.1	Magmatic	122	42	8.1	0.25	0.01	0.35	0.5898	0.0111	0.0766	0.0012	0.62	0.0558	0.0005	0.0001	0.0000	0.0239	0.0005	471	7	**476**	**7**	446	22	-1.1	
EHS-09.1	Magmatic	590	223	39.3	0.06	-0.03	0.39	0.5914	0.0197	0.0770	0.0025	0.71	0.0557	0.0002	0.0000	0.0000	0.0238	0.0008	472	13	**478**	**15**	440	10	-1.4	
EJ50-06.1	Magmatic	349	113	23.4	0.32	0.05	0.33	0.6019	0.0102	0.0775	0.0013	0.70	0.0563	0.0002	0.0002	0.0001	0.0244	0.0004	478	6	**481**	**8**	464	10	-0.6	
EJ67-13.1	Magmatic	335	37	22.5	0.23	-0.02	0.11	0.5975	0.0157	0.0776	0.0020	0.70	0.0559	0.0003	0.0001	0.0000	0.0239	0.0007	476	10	**482**	**12**	447	12	-1.2	
EJ67-19.1	Magmatic	1651	239	111.4	0.09	0.07	0.15	0.5969	0.0102	0.0780	0.0013	0.70	0.0555	0.0002	0.0001	0.0000	0.0250	0.0006	475	7	**484**	**8**	433	8	-1.8	
EJ50-26.1	Magmatic	800	146	54.4	0.10	0.08	0.19	0.6112	0.0078	0.0786	0.0008	0.60	0.0564	0.0004	0.0001	0.0000	0.0251	0.0008	484	5	**488**	**5**	469	15	-0.7	
EJ50-29.1	Magmatic	2477	80	168.6	0.05	-0.02	0.03	0.6106	0.0096	0.0786	0.0012	0.71	0.0563	0.0002	0.0000	0.0000	0.0237	0.0005	484	6	**488**	**7**	465	7	-0.8	
EHS-42.1	Recrsytallised	279	2	19.1	0.27	0.04	0.01	0.6088	0.0027	0.0790	0.0004	0.72	0.0559	0.0000	0.0001	0.0000	0.0361	0.0002	483	2	**490**	**2**	448	0	-1.5	
EHS-02.1	Magmatic	571	55	39.1	0.23	0.13	0.10	0.6036	0.0137	0.0791	0.0017	0.70	0.0553	0.0003	0.0001	0.0000	0.0267	0.0007	480	9	**491**	**10**	426	12	-2.3	
EJ50-25.1	Magmatic	507	166	34.9	0.19	0.09	0.34	0.6151	0.0029	0.0796	0.0004	0.72	0.0561	0.0000	0.0001	0.0000	0.0252	0.0001	487	2	**494**	**2**	455	0	-1.4	
EJ67-16.1	Magmatic	233	51	16.1	0.16	-0.04	0.23	0.6088	0.0196	0.0800	0.0025	0.71	0.0552	0.0003	0.0001	0.0001	0.0246	0.0009	483	12	**496**	**15**	419	10	-2.8	
EJ50-09.1	Magmatic	532	198	37.2	0.23	-0.16	0.38	0.6146	0.0091	0.0807	0.0011	0.67	0.0552	0.0003	0.0001	0.0000	0.0244	0.0004	487	6	**500**	**7**	421	12	-2.9	
EHS-41.1	Magmatic	451	181	35.9	0.16	0.30	0.41	0.7537	0.0116	0.0921	0.0014	0.72	0.0594	0.0000	0.0001	0.0000	0.0298	0.0005	570	7	**568**	**8**	580	0	0.4	
EHS-36.1	Magmatic	372	330	30.2	0.10	-0.02	0.91	0.7848	0.0176	0.0937	0.0020	0.70	0.0607	0.0003	0.0001	0.0000	0.0290	0.0006	588	10	**578**	**12**	630	11	1.8	
EJ67-40.1	Recrsytallised	200	595	16.8	0.21	-1.51	3.05	0.8160	0.0207	0.0973	0.0023	0.67	0.0608	0.0006	0.0001	0.0000	0.0295	0.0007	606	12	**599**	**14**	633	21	1.2	
EHS-21.1	Magmatic	851	655	71.9	0.07	-0.12	0.79	0.7963	0.0146	0.0976	0.0018	0.71	0.0592	0.0002	0.0000	0.0000	0.0299	0.0006	595	8	**600**	**10**	574	6	-0.9	
EJ50-39.1	Recrsytallised	209	113	17.6	0.21	-0.15	0.56	0.8418	0.0083	0.0976	0.0010	0.72	0.0626	0.0000	0.0001	0.0000	0.0296	0.0003	620	5	**600**	**6**	694	0	3.2	
EHS-40.1	Magmatic	785	905	69.5	0.17	0.20	1.18	0.8678	0.0099	0.1023	0.0012	0.72	0.0615	0.0000	0.0001	0.0000	0.0319	0.0004	634	5	**628**	**7**	658	0	1.0	
EJ50-23.2	Magmatic	128	179	11.4	-0.08	-0.30	1.43	0.8446	0.0232	0.1026	0.0026	0.66	0.0597	0.0006	0.0000	0.0001	0.0313	0.0008	622	13	**630**	**15**	593	23	-1.3	
EHS-13.1	Magmatic	448	577	40.0	0.23	-0.53	1.32	0.8532	0.0349	0.1031	0.0042	0.72	0.0600	0.0003	0.0001	0.0000	0.0311	0.0013	626	19	**633**	**25**	604	9	-1.0	
EJ67-14.1	Magmatic	204	101	18.6	0.25	-0.11	0.50	0.8661	0.0231	0.1053	0.0028	0.71	0.0597	0.0002	0.0001	0.0000	0.0320	0.0010	633	13	**645**	**16**	592	8	-1.9	
EHS-35.1	Magmatic	275	314	26.3	0.23	0.38	1.17	0.9519	0.0272	0.1104	0.0031	0.72	0.0625	0.0001	0.0001	0.0000	0.0346	0.0010	679	14	**675**	**18**	693	5	0.6	
EJ67-26.1	Magmatic	384	176	36.8	0.06	-0.22	0.47	0.9540	0.0278	0.1108	0.0032	0.71	0.0624	0.0003	0.0000	0.0000	0.0331	0.0012	680	15	**678**	**18**	689	12	0.4	
EHS-24.1	Magmatic	221	116	21.9	0.09	0.09	0.54	0.9969	0.0302	0.1141	0.0034	0.70	0.0634	0.0004	0.0000	0.0000	0.0355	0.0011	702	15	**697**	**20**	720	13	0.8	
EJ67-23.1	Recrsytallised	289	100	28.6	0.10	-0.06	0.35	0.9588	0.0277	0.1143	0.0033	0.71	0.0608	0.0003	0.0001	0.0000	0.0347	0.0011	683	15	**698**	**19**	634	11	-2.2	
EJ67-22.1	Metamorphic	385	7	39.0	0.05	0.03	0.02	1.0502	0.0217	0.1171	0.0023	0.68	0.0651	0.0004	0.0000	0.0000	0.0399	0.0013	729	11	**714**	**13**	777	14	2.1	
EHS-29.1	Recrsytallised	210	18	22.4	0.02	0.04	0.09	1.0868	0.0237	0.1232	0.0027	0.72	0.0640	0.0001	0.0000	0.0000	0.0390	0.0010	747	12	**749**	**15**	741	4	-0.3	
EHS-08.1	Magmatic	1410	661	154.2	0.01	-0.23	0.48	1.0965	0.0419	0.1263	0.0048	0.72	0.0630	0.0002	0.0000	0.0000	0.0375	0.0015	752	21	**767**	**28**	707	8	-2.0	
EJ67-21.1	Magmatic	126	72	13.9	0.18	-0.05	0.59	1.1436	0.0344	0.1277	0.0037	0.70	0.0649	0.0005	0.0001	0.0001	0.0389	0.0012	774	16	**775**	**21**	773	15	-0.1	
EJ67-06.1	Magmatic	357	393	40.0	0.03	0.09	1.13	1.1476	0.0262	0.1294	0.0029	0.70	0.0643	0.0003	0.0000	0.0000	0.0398	0.0011	776	12	**785**	**16**	752	11	-1.1	
EJ67-33.1	Magmatic	420	721	47.2	0.04	0.14	1.76	1.1671	0.0290	0.1297	0.0032	0.71	0.0653	0.0002	0.0000	0.0000	0.0398	0.0011	785	14	**786**	**18**	783	8	-0.1	
EJ50-24.2	Uncertain	780	275	92.4	0.00	-0.07	0.36	1.2318	0.0446	0.1368	0.0050	0.72	0.0653	0.0001	0.0000	0.0000	0.0412	0.0015	815	21	**827**	**28**	784	3	-1.4	
EJ67-22.2	Recrsytallised	576	131	161.3	0.04	0.21	0.23	5.3488	0.3598	0.3235	0.0202	0.67	0.1199	0.0030	0.0000	0.0000	0.0995	0.0064	1877	59	1807	99	**1955**	**43**	7.6	
EJ67-21.2	Uncertain	45	20	14.4	0.22	0.01	0.44	6.4172	0.2467	0.3676	0.0133	0.68	0.1266	0.0017	0.0001	0.0001	0.1051	0.0043	2035	34	2018	63	**2052**	**23**	1.6	
EJ67-32.1	Recrsytallised	123	29	50.9	0.02	0.02	0.24	11.2040	0.2785	0.4800	0.0118	0.71	0.1693	0.0007	0.0000	0.0000	0.1340	0.0047	2540	24	2527	52	**2551**	**6**	0.9	

Discordant analyses (excluded)

EJ50-30.1	Magmatic	673	404	3.0	6.42	4.72	0.62	0.0584	0.0015	0.0052	0.0001	0.69	0.0810	0.0006	0.0034	0.0002	0.0024	0.0001	58	1	**34**	**1**	1221	15	41.7	Discordant
EJ50-20.1	Magmatic	653	283	3.2	7.48	0.39	0.44	0.0410	0.0017	0.0057	0.0002	0.70	0.0521	0.0006	0.0040	0.0006	0.0019	0.0001	41	2	**37**	**2**	288	24	10.0	Discordant
EJ67-03.2	Magmatic	869	374	4.6	0.78	932.03	0.44	0.8764	0.0331	0.0062	0.0002	0.71	1.0305	0.0065	0.0004	0.0001	0.2559	0.0098	639	18	**40**	**1**	5280	9	93.8	Discordant
EJ67-35.1	Magmatic	666	281	3.7	0.15	1176.33	0.43	0.4316	0.0108	0.0064	0.0002	0.70	0.4902	0.0027	0.0001	0.0003	0.3407	0.0093	364	8	**41**	**1**	4212	8	88.7	Discordant
EJ67-28.1	Magmatic	706	174	4.0	1.67	1042.76	0.25	0.5423	0.0162	0.0065	0.0002	0.60	0.6057	0.0099	0.0009	0.0002	0.5398	0.0178	440	11	**42**	**1**	4522	23	90.5	Discordant
EJ67-39.1	Magmatic	509	395	3.5	-0.21	1233.15	0.80	0.6316	0.0182	0.0079	0.0002	0.69	0.5811	0.0049	-0.0001	0.0002	0.2260	0.0068	497	11	**51**	**1**	4462	12	89.8	Discordant
EJ50-02.2	Magmatic	568	193	3.9	1.70	1074.03	0.35	0.4261	0.0425	0.0080	0.0002	0.15	0.3855	0.0376	0.0009	0.0001	0.4899	0.0581	360	31	**52**	**1**	3854	140	85.7	Discordant
EJ50-05.1	Magmatic	497	398	3.7	0.30	1132.34	0.82	0.5498	0.0504	0.0085	0.0002	0.21	0.4670	0.0409	0.0002	0.0002	0.2168	0.0243	445	34	**55**	**2**	4140	124	87.7	Discordant
EJ50-34.1	Magmatic	436	186	3.3	0.97	1311.44	0.44	0.3531	0.0104	0.0087	0.0002	0.54	0.2938	0.0057	0.0005	0.0002	0.5103	0.0117	307	8	**56**	**1**	3438	30	81.8	Discordant
EJ50-07.2	Magmatic	406	193	3.1	0.96	1131.15	0.49	0.3230	0.0307	0.0088	0.0002	0.18	0.2670	0.0246	0.0005	0.0000	0.3963	0.0413	284	24	**56**	**1**	3289	138	80.2	Discordant
EJ67-09.1	Magmatic	445	210	3.4	1.25	1120.51	0.48	0.5464	0.0517	0.0088	0.0003	0.25	0.4505	0.0400	0.0007	0.0003	0.3961	0.0472	443	35	**57**	**2**	4087	126	87.2	Discordant
EJ67-01.2	Magmatic	519	301	4.0	0.39	1008.70	0.59	0.8276	0.0715	0.0089	0.0001	0.10	0.6737	0.0576	0.0002	0.0002	0.2892	0.0314	612	41	**57**	**1**	4676	118	90.7	Discordant
EJ50-10.1	Magmatic	403	203	3.1	-1.01	1205.69	0.52	0.3224	0.0300	0.0090	0.0005	0.41	0.2595	0.0198	-0.0005	0.0001	0.4058	0.0479	284	23	**58**	**3**	3244	115	79.6	Discordant
EJ67-20.2	Magmatic	389	176	3.1	0.62	1195.05	0.46	0.3246	0.0276	0.0092	0.0003	0.26	0.2566	0.0204	0.0003	0.0003	0.4614	0.0488	285	21	**59**	**2**	3226	120	79.4	Discordant
EHS-20.1	Magmatic	439	338	3.6	1.44	1171.24	0.79	0.5261	0.0518	0.0094	0.0005	0.42	0.4065	0.0325	0.0008	0.0001	0.2579	0.0301	429	35	**60**	**3**	3933	115	86.0	Discordant
EJ67-10.2	Magmatic	366	162	3.0	-0.01	1119.60	0.45	0.4764	0.0394	0.0095	0.0003	0.26	0.3637	0.0281	0.0000	0.0001	0.4571	0.0456	396	27	**61**	**2**	3766	112	84.6	Discordant
EJ50-15.2	Magmatic	363	166	3.0	1.47	1250.23	0.47	0.4041	0.0085	0.0096	0.0002	0.67	0.3046	0.0022	0.0008	0.0003	0.4994	0.0139	345	6	**62**	**1**	3494	11	82.1	Discordant
EJ50-32.1	Magmatic	390	171	3.3	1.02	1302.93	0.45	0.3489	0.0152	0.0097	0.0004	0.68	0.2605	0.0037	0.0005	0.0003	0.5511	0.0230	304	12	**62**	**3**	3250	22	79.5	Discordant
EHS-25.1	Magmatic	302	126	2.5	-0.54	965.90	0.43	0.6084	0.1510	0.0097	0.0020	0.58	0.4540	0.0663	-0.0003	0.0006	0.4296	0.1147	483	100	**62**	**12**	4099	201	87.1	Discordant, error > 10 %
EJ50-31.1	Magmatic	344	205	3.2	1.24	1157.56	0.61	0.5303	0.0453	0.0109	0.0001	0.11	0.3534	0.0298	0.0007	0.0003	0.3922	0.0394	432	31	**70**	**1**	3722	123	83.8	Discordant
EJ50-13.2	Magmatic	309	200	3.0	1.85	1101.40	0.66	0.7356	0.0654	0.0113	0.0005	0.36	0.4712	0.0362	0.0010	0.0003	0.3548	0.0375	560	39	**73**	**3**	4154	109	87.0	Discordant
EJ50-35.1	Magmatic	309	159	3.2	1.45	1242.09	0.53	0.6529	0.0232	0.0118	0.0004	0.71	0.4007	0.0021	0.0008	0.0001	0.5347	0.0193	510	14	**76**	**3**	3912	8	85.2	Discordant
EJ50-03.1	Magmatic	290	144	3.1	0.27	1296.89	0.51	0.5950	0.0247	0.0123	0.0005	0.71	0.3509	0.0024	0.0001	0.0002	0.6036	0.0252	474	16	**79**	**3**	3711	10	83.4	Discordant
EJ67-04.2	Magmatic	300	105	3.2	1.42	1158.08	0.36	0.4184	0.0340	0.0124	0.0002	0.17	0.2448	0.0193	0.0008	0.0002	0.7896	0.0754	355	25	**79**	**2**	3151	120	77.6	Discordant
EJ67-27.1	Magmatic	322	165	3.6	0.57	1062.24	0.53	0.9632	0.0746	0.0128	0.0004	0.31	0.5458	0.0382	0.0003	0.0002	0.4991	0.0478	685	39	**82**	**3**	4370	99	88.0	Discordant
EJ67-02.2	Magmatic	241	82	3.1	1.03	1135.40	0.35	0.6814	0.0510	0.0149	0.0006	0.38	0.3306	0.0210	0.0005	0.0002	0.9627	0.0895	528	31	**96**	**4**	3620	94	81.9	Discordant
EJ67-36.1	Recrsytallised	234	92	3.1	0.16	1164.78	0.40	1.0018	0.0267	0.0152	0.0004	0.71	0.4791	0.0023	0.0001	0.0003	0.8625	0.0240	705	14	**97**	**3**	4178	7	86.2	Discordant
EJ50-22.2	Magmatic	241	130	3.3	1.10	1282.67	0.55	0.7327	0.0271	0.0156	0.0005	0.67	0.3399	0.0044	0.0006	0.0002	0.6941	0.0260	558	16	**100**	**3**	3662	20	82.1	Discordant
EJ50-16.2	Magmatic	228	134	3.2	0.65	1089.78	0.61	1.0604	0.0728	0.0163	0.0004	0.25	0.4708	0.0303	0.0003	0.0003	0.5603	0.0478	734	37	**104**	**2**	4152	92	85.8	Discordant
EJ50-17.2	Magmatic	209	96	3.0	1.71	1279.20	0.47	0.6277	0.0068	0.0164	0.0002	0.61	0.2769	0.0016	0.0009	0.0001	0.8680	0.0131	495	4	**105**	**1**	3345	9	78.8	Discordant
EJ50-33.1	Magmatic	215	129	3.4	0.85	1115.94	0.62	1.1382	0.0724	0.0183	0.0006	0.34	0.4502	0.0252	0.0005	0.0003	0.6311	0.0531	772	35	**117**	**4**	4086	81	84.8	Discordant
EJ50-28.1	Recrsytallised	186	81	3.0	1.56	1297.34	0.44	0.6137	0.0182	0.0185	0.0004	0.56	0.2412	0.0045	0.0008	0.0003	1.0489	0.0290	486	12	**118**	**3**	3128	30	75.7	Discordant
EJ50-42.1	Magmatic	187	456	3.2	0.67	1547.31	2.50	1.9761	0.0775	0.0197	0.0007	0.66	0.7278	0.0112	0.0004	0.0001	0.1576	0.0061	1107	27	**126**	**5**	4787	22	88.6	Discordant
EJ50-08.1	Magmatic	189	124	3.5	1.66	1273.33	0.67	1.1649	0.0556	0.0213	0.0010	0.71	0.3967	0.0029	0.0009	0.0004	0.7582	0.0360	784	27	**136**	**6**	3897	11	82.7	Discordant
EJ67-34.1	Recrsytallised	203	101	6.4	3.18	4.63	0.51	0.4021	0.0439	0.0366	0.0026	0.47	0.0796	0.0066	0.0017	0.0006	0.0180	0.0018	343	32	**232**	**16**	1188	156	32.5	Discordant
EHS-06.1	Recrsytallised	1613	429	60.5	0.96	1.18	0.27	0.3548	0.0180	0.0433	0.0022	0.71	0.0594	0.0005	0.0005	0.0001	0.0174	0.0009	308	14	**274**	**13**	581	19	11.3	Discordant

Errors are at 1 σ level. The error in 206/238 averaging the standard has already been propagated. Th/U ratios are atomic (232/238), not in weight.

Point-to point errors, calculated on replicates of the TEMORA standard, are: 0.39 % for $^{206}Pb/^{238}U$, and 0.48 % for $^{207}Pb/^{206}Pb$

[1]f206_4 and f206_8 are the fractions of common ^{206}Pb with regard to total ^{206}Pb, calculated based on measured ^{204}Pb and ^{208}Pb, respectively.

[2]The preferred ages are in bold numbers (and highlighted in green). For zircons younger than 1.5 Ga, the $^{206}Pb/^{238}U$ age is preferred, and the discordance is calculated Disc. (%) = ($^{206}Pb/^{238}U$ age / $^{207}Pb/^{235}$ U age) x 100. For zircons older than 1.5 Ga, the $^{207}Pb/^{206}Pb$ age is preferred, and the discordance is calculated Disc. (%) = ($^{206}Pb/^{238}U$ age / $^{207}Pb/^{206}Pb$ age) x 100.

Kernel Density Estimates (KDE) plots for the whole age spectrum for the three studied APM samples.

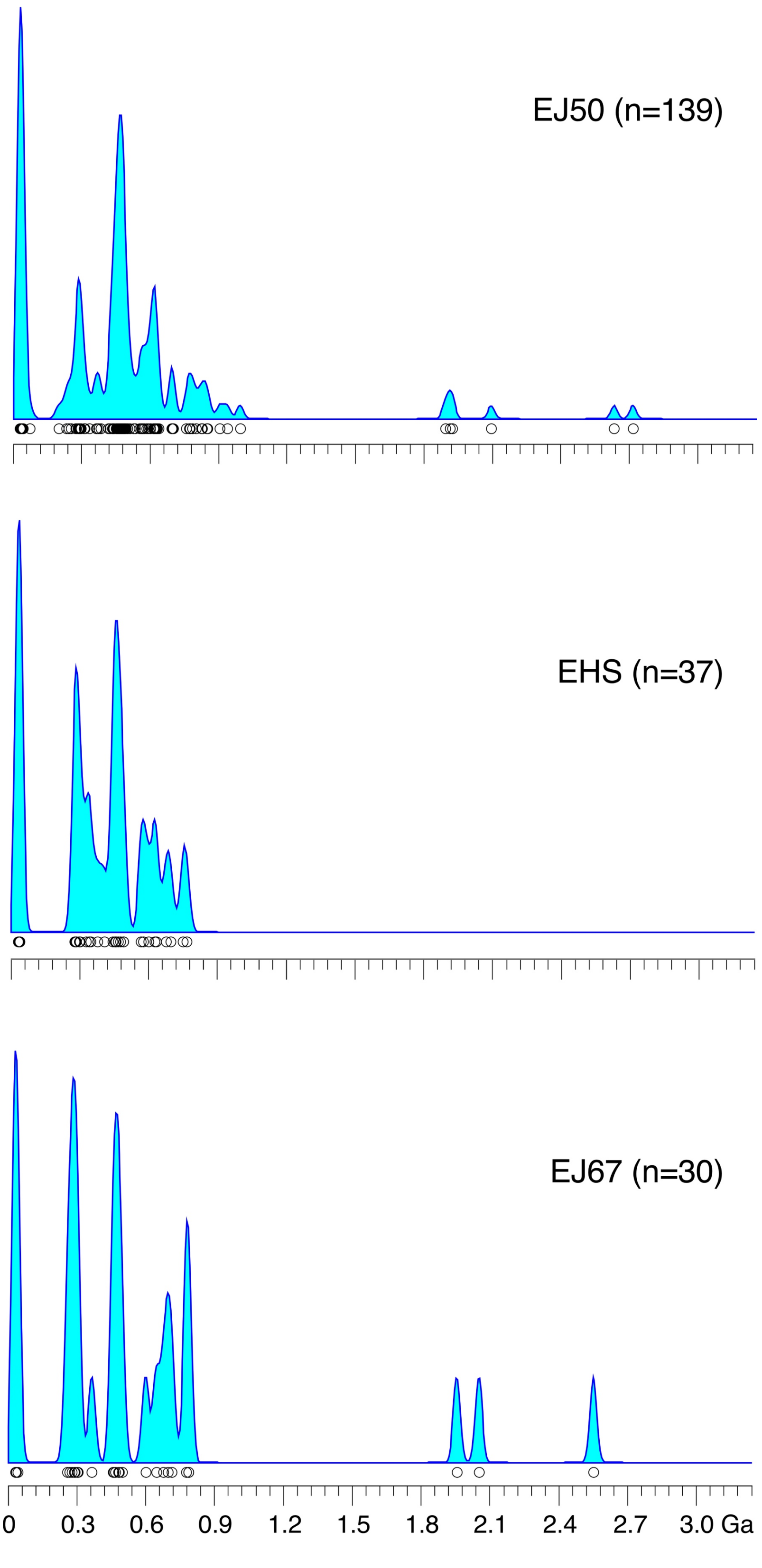

Supplementary file 3d: Lu-Hf data

Sample	Nr.	Age(Ma)	176Hf/177Hf	1s	176Lu/177Hf	1s	176Yb/177Hf	1s	177/176t	2s	eHf(t)	2s	tDM	2s	Protolith age
4569-EJ50-19.1	EJ50	31	0.282731	0.000016	0.001511	0.000037	0.046474	0.000762	0.282730125	3.2E-05	-1.25	1.13	0.74	0.02	1.14
4571-EJ50-19.2	EJ50	31	0.282705	0.000016	0.001052	0.000012	0.031374	0.000382	0.282704391	3.2E-05	-2.16	1.13	0.77	0.02	1.20
4561_EJ50-27.1	EJ50	33	0.282708	0.000026	0.002804	0.000018	0.082404	0.000459	0.282706272	5.2E-05	-2.05	1.84	0.80	0.04	1.19
4563_EJ50-27.2	EJ50	33	0.282693	0.000018	0.001454	0.000017	0.04989	0.000471	0.282692104	3.6E-05	-2.55	1.27	0.79	0.03	1.22
4573-EJ50-36.1	EJ50	31.9	0.282678	0.000018	0.000971	0.000008	0.028172	0.000108	0.282677422	3.6E-05	-3.10	1.27	0.81	0.02	1.26
4575_EJ50-36.2	EJ50	32	0.282716	0.000022	0.00128	0.000015	0.03511	0.000284	0.282715235	4.4E-05	-1.76	1.56	0.76	0.03	1.17
4577_EJ50-20.1	EJ50	34.3	0.282661	0.000019	0.001372	0.000015	0.042791	0.000117	0.282660121	3.8E-05	-3.66	1.34	0.84	0.03	1.29
4581_EJ50-30.1	EJ50	31.2	0.282695	0.00002	0.001854	0.000042	0.054475	0.000892	0.28269392	4E-05	-2.53	1.41	0.80	0.03	1.22
4603_EJ67-7.1	EJ67	34.2	0.282667	0.000016	0.002362	0.000041	0.075246	0.000813	0.282665491	3.2E-05	-3.47	1.13	0.85	0.02	1.28
4604_EJ67-7.2	EJ67	34.2	0.282642	0.000014	0.001597	0.000015	0.052875	0.000566	0.28264098	2.8E-05	-4.33	0.99	0.87	0.02	1.33
4607_EJ67-24.1	EJ67	39.8	0.282801	0.000011	0.001674	0.00003	0.05109	0.000622	0.282799756	2.2E-05	1.41	0.78	0.65	0.02	0.98
4608_EJ67-24.2	EJ67	39.8	0.282763	0.000019	0.001725	0.000012	0.046227	0.000402	0.282761718	3.8E-05	0.06	1.34	0.70	0.03	1.07
4605_EJ67-25.1	EJ67	31.2	0.282623	0.000011	0.000777	0.00002	0.022776	0.000481	0.282622547	2.2E-05	-5.05	0.78	0.88	0.01	1.38
4606_EJ67-25.2	EJ67	31.2	0.282578	0.000011	0.000409	0.000003	0.013133	0.00004	0.282577762	2.2E-05	-6.64	0.78	0.93	0.02	1.47
4629_EJ67-38.1	EJ67	30.7	0.282721	0.00001	0.001462	0.00002	0.047019	0.000398	0.282720162	2E-05	-1.61	0.71	0.75	0.01	1.16
4643_EHS11-3.1	EHS11	36.9	0.282657	0.00001	0.000932	0.000005	0.030151	0.000121	0.282656358	2E-05	-3.73	0.71	0.83	0.01	1.30
4646_EHS11-5.1	EHS11	29.4	0.282645	0.000012	0.001222	0.000008	0.040509	0.000188	0.282644329	2.4E-05	-4.32	0.85	0.86	0.02	1.33
4659_EHS11-5.2	EHS11	36.2	0.282655	0.000011	0.00128	0.000014	0.041772	0.000408	0.282654135	2.2E-05	-3.82	0.78	0.84	0.02	1.30
4654_EHS11-14	EHS11	38.2	0.282613	0.000017	0.001346	0.000009	0.040941	0.000226	0.28261204	3.4E-05	-5.27	1.20	0.90	0.02	1.40
4647_Ehs11-26.1	EHS11	31.3	0.282638	0.000011	0.001219	0.000021	0.040332	0.000685	0.282637287	2.2E-05	-4.53	0.78	0.87	0.01	1.34
4656_EHS11-33.1	EHS11	38.6	0.282736	0.000014	0.001363	0.00001	0.0392	0.000476	0.282735017	2.8E-05	-0.91	0.99	0.73	0.02	1.12
4657_EHS11-34.1	EHS11	35.2	0.282621	0.000015	0.000939	0.000006	0.028307	0.000041	0.282620383	3E-05	-5.04	1.06	0.88	0.02	1.38

Supplementary File 4: Age-map of the potential source areas supplying the Cenozoic zircons to the Acquadolce phyllites and metasiltstones (APM)

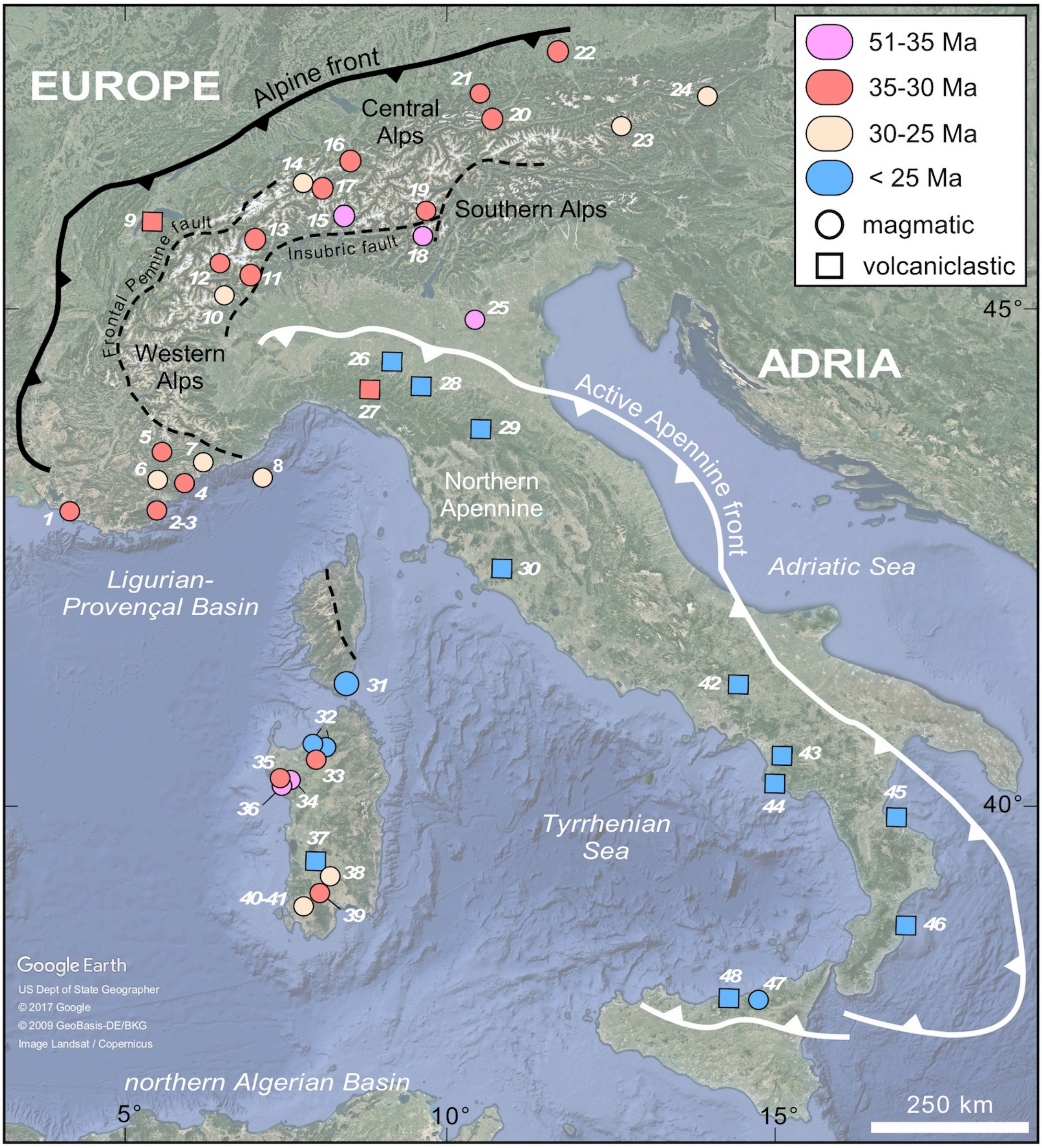

Fig. S4a: Distribution of potential source rocks supplying the Cenozoic zircons to the Acquadolce phyllites and metasiltstones (APM). In the map are shown both in situ magmatic rocks (circle) and volcaniclastics rocks (square) with ages between ~50 and ~25 Ma. Different colours correspond to different ages. Basemap from Google Earth, other artwork created using Canvas draw 3 (version 3.0.3) http://www.canvasgfx.com/en/products/canvas-draw

Sector	Locality	Formation	Age (Ma)	Method	Rock type	Reference	Figura
Provence							
	Esterel, France south-eastern border of the European plate	Estrellite du Drammont	31.9±0.7 Ma;	K-Ar	pebbles and volcaniclastics andesitic-dacitic	Montenat et al., 1999	2
			32.7±0.9 Ma	K-Ar		Ivaldi et al., 2003	3
		Gres de Champsaur	32.5±2.2 Ma	Ar-Ar	andesitic pebbles	Feraud et al., 1995	1
		Tourette sur Loup	32.5-30.8	K-Ar	andesitic volcanites	Ivaldi et al., 2003; Feraud et al., 1995	5
		Juan-Les-Pin	32.5-30.8	K-Ar			4
		Vanade	32.5-30.8	K-Ar			
		Biot; Grasse	26.2	K-Ar			6
		Gres de Tavayanne	31.0±0.4; 32.0±0.4 Ma	Ar-Ar	andesitic pebbles	Ruffini et al. 1997	9
		Dacite de Baratus	29.6±0.5; 29.0±0.5 Ma	Ar-Ar	andesitic pebbles	Feraud et al., 1995	
		Cap d'Ail	27.8 Ma	K-Ar	andesitic volcanites	Ivaldi et al., 2003	7
		Ligurian-Provencial sea	30-12	K-Ar	andesite	Ivaldi et al., 2003	8
Sardinia-Corsica							
**Sardinia**		Alghero	28.6±1.5	K-Ar	diorite	Giraud et al., 1979	36
		Valverde	31.5/35.3±1.5	K-Ar	porphirites	Montigny et al., 1981	35
		Turriga	22/21±1	K-Ar	volcanites	Savelli et al., 1975	39
		Osilo	31.2±1.1	K-Ar	andesite	Montigny et al., 1981	34
		Siliqua	32.1±0.9	K-Ar	andesite	Montigny et al., 1981	40
main references in Beccaluva et al., 1985; Lustrino et al., 2009	Sardinia-Corsica volcanic Arc	Castelsardo	19.7/18.5±0.5	K-Ar	volcanite	Savelli et al., 1975	33
		Anglona	20.9/18.9±0.7	K-Ar	volcanites	Beccaluva et al.,1985; 2015	
		Villanovaforru	Burdigalian	stratigraphic	porphyric, doleritic	Bruguier et al., 2009	38
		Perdaxius	26.3/22.8±0.9	K-Ar	dacite	Montigny et al., 1981	41
		Acquafredda	27.8±1	K-Ar	dacite	Savelli et al., 1975	
		Calabona	38 Ma	Ar-Ar, San	microdiorite	Lustrino et al., 2009	37
**Corsica**		Francolu	19.9±0.7	K-Ar	rhyolitic tuff	Ottaviani-Spella et al., 2001	32
		Tre Paduli	18.9-19.3±0.5	K-Ar	dacitic ignimbritic tuff	Ottaviani-Spella et al., 2001	
		Balistra	19.2±0.5	K-Ar	rhyolitic tuff	Ottaviani-Spella et al., 2001	
Alpine Periadriatic							
**Western Alps**	Sesia-Lanzo Zone	Traversella Pluton	31±1	K-Ar, Bt	diorite	Krummenacher et al., 1960	10
			30±1	K-Ar, Bt	Sienite	Krummenacher et al., 1960	
		Valle del Cervo Pluton	30.40±0.05	U-Pb, Zrn	granitoid	Kapferer et al. 2009	12
			31±0.2	U-Pb, Zrn	granitoid	Romer et al., 1996	
			31-30±0.5	Rb-Sr, Bt	monzonite	Bigioggero et al., 1994	
			29±0.5	Rb-Sr, Bt	granitoid		
		Migliano	33.00±0.04	U-Pb, Zrn	tonalite	Berger et al. 2012	12
			33-29	Rb-Sr, K-Ar Bt	tonalite	Carraro & Ferrara, 1968	
		Lamproitic dyke	30.3-32.7	K-Ar/Rb-Sr, Bt	lamproite	Dal Piaz, 1973; Petke et al., 1997	13
			31.7±0.2; 32.4±0.5	U-Pb, Ttn	granodiorite-porphyrite vein	Romer et al., 1996	
		Volcanic rocks	32.65±0.24	U-Pb, Zrn	tonalitic	Kapfere et al., 2009	11
	Periadriatic–Sava–Vardar magmatic belt	Rhaetian Alps	32.4-30.8	U-Pb, Zrn	basalt, basaltic andesite, andesite	Dal Piaz et al., 1988	16
**Central Alps**	Bergell area	Bregaglia Pluton	32.4±0.4 (28.9-32.4)	U-Pb: Aln, Ttn, Zrn, Ap	gabbro-tonalite	Gregory et al., 2009; von Blanckenburg et al., 1992	15
		Novate	26-24	U-Pb, Mnz, Zrn	granite, monzonite, syenite	Liati et al., 2000	14
	Presolana- Gandino area	Presolana- Gandino dykes and stock	34-42	U-Pb, Zrn	andesite	Bergomi et al., 2016; D'Adda et al., 2011	17
**Eastern Alps**	Adamello (~42-29)	Avio	35.2-30.5	U-Pb, Zrn	quarzodiorite	Del Moro et al., 1983	18
		Redicastello	42.6-36.1	U-Pb: Zrn, Ttn	granodiorite-tonalite	Del Moro et al., 1983; Shoene et al., 2012; Shalteggger et al., 2009	
		Presanelle	36.3-32.0	U/Pb, Zrn	tonalite	Del Moro et al., 1983; Mayer et al., 2003;	
**South-Western Tyrol**	Ortles-Cevedale area	Dykes and stocks	32-30	Rb-Sr, Bt-wr	quarzo-dioritic stock	Dal Piaz et al., 1988	19
			31.9	U-Pb, Zrn	dyke	Muller et al., 2001	
			32-28	Rb-Sr, Bt	lamellae	Martin et al., 1993	
	Vedrette di Ries	Vedrette di Ries pluton	31.8-32.2	U-Pb, Aln	granite, tonalite	Romer et al., 2003	21
			31	(Rb-Sr, wr)		Borsi et al., 1979	
	Cima di Villa	Cima di Villa pluton	29.5	Rb/Sr, wr-Bt	not reported	Borsi et al., 1979	20
		Tauren window	32.5-31	U-Pb, Zrn	shoshonitic dyke	Muller et al., 2000; 2001	22
	Periadriatic–Sava–Vardar magmatic belt	Karavanke pluton	28-30	U-Pb, Zrn	tonalite, granodiorite, diorite, gabbro	Kovacs et al., 2007	23
		Pohorje intrusion	28-31	U-Pb, Zrn		Pamić and Palinkaš, 2000	24
**Veneto province**	Veneto	VVP	30-52	U-Pb, Zrn	basalts	Visoná et al., 2007	25
Volcaniclastic Levels							
**Northern-Central Italy**	Mortara	Northern Apennine	32-29	biostratigraphic	calcalkaline volcanites	Mattioli et al., 2002; Mattioli et al., 2012	26
	Ranzano Fm.	Sub-Ligure	32-30	Ar-Ar	andesitic dacite volcanoclast	Cibin et al., 2001; Mattioli et al., 2002	27
	Antognola	Epi-Ligure	Burdigalian	biostratigraphic		Critelli et al., 1993; De Capoa et al., 2002	
	Aveto-Petrignacola	Sub-Ligure	29-32	Ar-Ar; U-Pb, Zrn	lava clast	Anfinson et al., 2016; Mattioli et al., 2002; Malusà et al., 2013	28
	Cervarola	Tuscan Nappe	Late Oligo-Burdigalian	biostratigraphic	rhyolitic lava	Delle Rose et al., 1994	29
	Vicchio marls	Cervarola Unit-Tuscan Nappe	Late Aquitanian-Burdigalian	biostratigraphic	rhyolitic-dacitic fragments	Crisci et al., 1988; Critelli et al., 1993; De Capoa et al., 2002	30
	Bisciaro	Northern Apennine	26.8-17	Ar-Ar	andesite, rhyolite	Balogh et al., 1993; Guerrera et al., 2015	30
	Bagni Sant'Agostino	Tuscan Nappe	Burdigalian	biostratigraphic	basaltic-andesitic fragments	Guerrera et al., 2015	31
**Southern Italy**	Macchialupo	Daunia Complex	Burdigalian	biostratigraphic	lava clasts	Balogh et al., 1993	42
	Roccadaspite	Daunia Complex	Burdigalian	biostratigraphic	andesitic and/or basaltic fragments	Guerrera et al., 2015	43
	Pollica	Cilento group	Burdigalian-Langhian	biostratigraphic	rhyolitic–rhyodacitic fragment	Balogh et al., 1993	44
	Paludi	Peloritani Arc	late Olig-early miocene	biostratigraphic	basaltic-andesitic fragments	Guerrera et al., 2005	45
	Stilo-Capo d'Orlando	Peloritani Arc	late Olig-early miocene	biostratigraphic	lava clasts	Guerrera et al., 2005	46
	Tusa tufite	Sicilide complex	late Oligocene	biostratigraphic	andesite to dacite fragments	Guerrera et al., 2005	47
	Poggio Maria sandstone	Internal Magrebian Basin	late Oligocene	biostratigraphic	andesite fragments	Guerrera et al., 2015	48
	Valencia basin	Alboran-Valencia rift	Olig-Mio	biostratigraphic	andesite, rhyolite	Turner et al., 1999	49

Table S4: For each potential source rock are indicated locality, formation, age, dating method, rock type, reference and corresponding number on the age-map.

References

Anfinson, O.A., Malusà, M.G., Ottria, G., Dafov, L.N. & Stockli, D. F. Tracking coarse-grained gravity flows by LASS-ICP-MS depth-profiling of detrital zircon (Aveto Formation, Adriatic foredeep, Italy). Marine and Petroleum Geology 77, (2016)

Balogh, K., Delle Rose, M., Guerrera, F., Ravasz-Baranyai, L. & Veneri, F. New data concerning the inframiocenic "Bisciaro vocan- iclastic events" (Umbro–Marche Apennines) and comparison with similar occurrences. Giorn Geol 55, 83–104 (1993)

Beccaluva, L., Civetta, L., Macciotta, G. & Ricci, C.A. Geochronology in Sardinia: results and problems. Rend. Soc. Geol. It. 40, 57-72 (1985).

Berger, A., Thomsen, B.T., Ovtcharova, M., Kapferer, N. & Mercolli, I. Dating the emplacement and evolution of the orogenic mag- matism in the internal Western Alps: The Miagliano Pluton. Swiss J Geosci 105, 49–65 (2012)

Bigioggero, B,. Colombo, A., Del Moro, A., Gregnanin, A., Macera, P. & Tunesi A. The Oligocene Vallo del Cervo Pluton: an example of shoshonitic magmatism in the Western Italian Alps. Mem Sci Geol Padova 46, 409–421 (1994)

Borsi, S., Del Moro, A., Sassi, F.P. & Zirpoli, G. On the age of the Vedrette di Ries (Rieserferner) massif and its geodynamic sig- ni cance. Geol Rund 68, 41–60 (1979)

Bruguier, O., Hammor, D., Bosch, D. & Caby, R. Miocene incorporation of peridotite into the Hercynian basement of the Maghrebides (Edough massif, NE Algeria): Implications for the geodynamic evolution of the Western Mediterranean. Chemical Geology 261, 171–183 (2009)

Carraro, F., Ferrara, G. Alpine "tonalite" at Miagliano, Biella (Zona dioritico-kinzigitica): a preliminary note. Schweiz Miner Petrogr 48, 75–80 (1968)

Cibin, U., Spadafora, E., Zuffa, G. G. & Castellarin, A. Continental collision history from arenites of episutural basins in the Northern Apennines, Italy. Geological Society of America 113, 4-19 (2001).

Critelli, S. & Monaco, C. Depositi vulcanoclastici nell'unità del ysch Calabro-Lucano (Complesso Liguride, Appennino meridionale). Boll Soc Geol Ital 112, 121–132 (1993)

D'Adda, P., Zanchi, A., Bergomi, M., Berra, .F, Malusà, M.G., Tunesi, A. & Zanchetta, S. Polyphase thrusting and dyke emplacement in the central Southern Alps (Northern Italy). Int. J. Earth Sci. 100, 1095–1113 (2011)

Dal Piaz, G.V., Del Moro, A., Martin, S. & Venturelli, G. Post-col- lisional magmatism in the Ortler-Cevedale Massif (Northern Italy). Jahr Geol Bund 131, 533–551 (1988)

de Capoa, P., Di Staso, A., Guerrera, F., Perrone, V., Tramontana, M. & Zaghloul, M.N. The Lower Miocene volcaniclastic sedi- mentation in the Sicilian sector of the Maghrebian Flysch Basin: geodynamic implications. Geodin Acta 15:141–157 (2002)

Del Moro, A., Pardini, G.C., Quercioli, C., Villa, I.M. & Callegari, E. Rb/Sr and K/Ar chronology of Adamello granitoids, Southern Alps. Mem. Soc. Geol. It. 26, 285–299 (1983)

Delle Rose, M., Guerrera, F., Renzulli, A. & Serrano, F. Stratigraphic and volcaniclastic events in the Vicchio Marls Auct. (Cervarola Tectonic Unit) along the Monte Fatucchio section (northern Apennines, Italy). Giorn. Geol. 56, 97-114 (1994)

Féraud, G., Ruffet, G., Stéphan, J.F., Lapierre, H., Delgado, E. & Popoff, M.. Nouvelles données géochronologiques sur le volcanismepaléogène des Alpes occidentales: existence d'unévénement magmatique bref généralisé, in: Séance spéc. Soc.géol. France et Ass. Géologues Sud-Est,«Magmatismes dansle Sud-Est de la France», 38 (1995)

Giraud, J., Bellon, H. & Turco, G. L'intrusion microdioritique tertiaire d'Alghero (Sardaigne). Age K/Ar et relation avec le migmatisme calco-alcalin sarde. Analogies avec les esterellites de l'Esterel (Var.) C.R. Acad. Sc. Paris 288, 9-12 (!979)

Gregory, C.J., McFarlane, C.R.M., Hermann, J. & Rubatto, D. Tracing the evolution of calc-alkaline magmas: in-situ Sm–Nd isotope studies of accessory minerals in the Bergell Igneous Complex, Italy. Chem Geol 260, 73–86 (2009)

Guerrera, F., Martin-Martin, M., Perrone, V. & Tramontana, M. Tectono-sedimentary evolution of the southern branch of the West- ern Tethys (Maghrebian Flysch Basin and Lucanian Ocean). Terra Nova 17, 358–367 (2005)

Guerrera, F., Martín-Martín, M., Raffaelli, G. & Tramontana, M. The Early Miocene "Bisciaro volcaniclastic event" (northern Apennines, Italy): a key study for the geodynamic evolution of the central-western Mediterranean. International Journal of Earth Sciences 104 (2015).

Ivaldi, J.P., Bellon, H., Guardia,P., Mangan,C., Müller, C., Perez, J.L. & Terramorsi, S. Contexte lithostructural, âges 40K/40Ar et géochimie du volcanisme calco-alcalin tertiaire de Cap-d'Ail dans le tunnel ferroviaire de Monaco. Comptes Rendus Geoscience 335, 411-421 (2003).

Pamić, J. & Palinkas, L. Petrology and geochemistry of Paleogene tonalites from the easternmost parts of the Periadriatic Zone sepa- rating the Eastern Alps and the northwestern Dinarides, Mineral. Petrol. 70, 121–140 (2000).

Kapferer, N., Mercolli, I. & Berger, A. A unaltered paleosurface (regolith) preserved since Oligocene times on top of the Sesia Zone, Western Italian Alps. (2009).

Kovács, I.L., Csontos, L., Szabó, C.S., Bali, E., Falus, G.Y.,Benedek, K. & Zajacz, Z.. Paleogene–early Miocene igneous rocks and geodynamics of the Alpine-Carpathian-Pannonian-Dinaric region: An integrated approach. 418, 93-112 (2007)

Krummenacher, D. & Evernden, J. Détermination d'âge isotopique sur quelques roches des Alpes par la méthode K-Ar. Schweiz Miner Petrogr 40, 267–277 (1960)

Liati, A., Gebauer, D. & Fanning, M. U–Pb SHRIMP dating of zircon from the Novate granite (Bergell, Central Alps): evidence for Oligocene–Miocene magmatism, Jurassic/Cretaceous continental rifting and opening of the Valais trough. Schweiz Miner Petrogr 80, 305–316 (2000)

Lustrino, M., Morra, V., Fedele, L. & Franciosi, L. Beginning of the Apennine subduction system in central western Mediterranean: Constraints from Cenozoic "orogenic" magmatic activity of Sardinia, Italy. Tectonics 28 (2009).

Malusà, M.G., Carter, A., Limoncelli, M., Villa, I. M. & Garzanti, E. Bias in detrital zircon geochronology and thermochronometry. Chemical Geology 359, 90-107 (2013)

Martin, S., Prosser, G. & Morten, L. Tectono-magmatic evolution of sheeted plutonic bodies along the North Giudicarie Line (north- ern Italy). Geol. Rund. 82, 51–66 (1993)

MATTIOLI, M., BATTISTINI, G. D. & ZANZUCCHI, G. Geochemical features of the Tertiary buried Mortara volcanic body (Northern Apennines, Italy). Boll. Soc. Geol. lt. 1, 239-249 (2002).

Mattioli, M., Lustrino, M., Ronca, S. & Bianchini, G. Alpine subduction imprint in Apennine volcaniclastic rocks. Geochemical–petrographic constraints and geodynamic implications from Early Oligocene Aveto-Petrignacola Formation (N Italy). Lithos 134-135, 201-220 (2012)

Montenat, C., Leyrit, H., Gillot, P.Y., Janin, M.C. & Barrier, P. Extension du volcanisme oligocene dans l' arc de Castellane (chaines subalpines de Haute-Provence)Geologie de la France 1, 43-48 (1999).

Montigny, R., Edel, J.B. & Thuizat, R. Oligo-Miocene rotation of Sardinia: K-Ar ages and paleomagnetic data of Tertiary volcanics. Eartth and Planetary Science Letters, 54, 261-271 (1981)

Müller, W., Mancktelow, N.S. & Meier, M. Rb–Sr microchrons of synkinematic mica in mylonites: an example from the DAV fault of the Eastern Alps. Earth Planet. Sci. Lett. 180, 385–397 (2000)

Müller, W., Prosser, G., Mancktelow, N.S., Villa, I.M., Kelley, S.P., Viola, G. & Oberli, F. Geochronological constraints on the evolution of the Periadriatic Fault System. Int. J. Earth Sci. 90, 623–653 (2001)

Ottaviani-Spellaa, M.-M., Girarda, M., Rochette, P., Cheilletzc, A. & Thinone, M. Le volcanisme acide burdigalien du Sud de la Corse : pétrologie, datation K–Ar, paléomagnétisme. Earth and Planetary Sciences 333, 113-120 (2001).

Romer, R.L. & Siegesmund, S. Why allanite may swindle about its true age. Contrib Mineral Petrol 146, 297–307 (2003)

Romer, R.L., Scharer, U. & Steck, A. Alpine and pre-Alpine magmatism in the root-zone of the western central Alps. Contributions to Mineralogy and Petrology 123, 138-158 (1996).

Ruffini, R.O., Polino, R., Callegari, E., Hunziker, J.C. & Pfeifer, H.R. Volcanic clast rich turbidites of the Taveyanne sandstones from the Thones syncline (Savie, France): records for a Tertiary postcollisional volcanism. Schweiz Mineral Petrogr Mitt 77, 161–174 (1997)

Ruprecht, P. & Cooper, K. M. Integrating the uranium-series and elemental diffusion geochronometers in mixed magmas from Volcan Quizapu, Central Chile. J. Petrol. 53, 841–871 (2012).

Savelli, C. Datazioni preliminari col metodo K-Ar di vulcanici della Sardegna sud-occidentale. SIMP 31, 191-198 (1975).

Schaltegger, U., Brack, P., Ovtcharova, M., Peytcheva, I., Schoene, B., Stracke, A., Marocchi, M. & Bargossi G.M. Zircon ,and titanite recording 1.5 million years of magma accretion, crystallization and initial cooling in a composite pluton (southern Adamello batholith, northern Italy). Earth planet Sci Lett 286, 208–218 (2009)

Schoene B, Schaltegger U, Brack P, Latkoczy C, Stracke A, Günther D (2012) Rates of magma differentiation and emplacement in a ballooning pluton recorded by U–Pb TIMS-TEA, Adamello batholith, Italy. Earth Planet Sci Lett 355:162–173

Turner, S.P., Platt, J.P., George, R.M.M., Kelley, S.P., Pearson, D.G. & Nowell, G.M. Magma- tism associated with orogenic collapse of the Betic- Alboran domain SE Spain, Journal of Petrology, 40, 1011-1036 (1999)

Visonà, D., Caironi, V., Carraro, A., Dallai, L., Fioretti, A.M. & Fanning, M. Zircon megacrysts from basalts of the Venetian Volcanic Province (NE Italy): U–Pb ages, oxygen isotopes and REE data. Lithos 94, 168-180 (2007)

von Blanckenburg F. Combined high–precision chronometry and geochemical tracing using accessory minerals: applied to the Central-Alpine Bergell intrusion (central Europe). Chem. Geol. 100, 19–40 (1992)
